# Long non-coding RNA Lnc-408 promotes invasion and metastasis of breast cancer cell by regulating LIMK1

**DOI:** 10.1038/s41388-021-01845-y

**Published:** 2021-06-02

**Authors:** Yina Qiao, Ting Jin, Shengdong Guan, Shaojie Cheng, Siyang Wen, Huan Zeng, Maojia Zhao, Liping Yang, Xueying Wan, Yuxiang Qiu, Qiao Li, Manran Liu, Yixuan Hou

**Affiliations:** 1 Key Laboratory of Laboratory Medical Diagnostics, Chinese Ministry of Education, Chongqing Medical University, Chongqing, China; 2 Experimental Teaching & Lab Management Center, Chongqing Medical University, Chongqing, China

**Keywords:** Breast cancer, Non-coding RNAs

## Abstract

Invasion and metastasis are the leading causes of death in patients with breast cancer (BC), and epithelial-mesenchymal transformation (EMT) plays an essential role in this process. Here, we found that Lnc-408, a novel long noncoding RNA (lncRNA), is significantly upregulated in BC cells undergoing EMT and in BC tumor with lymphatic metastases compared with those without lymphatic metastases. Lnc-408 can enhance BC invasion and metastasis by regulating the expression of LIMK1. Mechanistically, Lnc-408 serves as a sponge for miR-654-5p to relieve the suppression of miR-654-5p on its target LIMK1. Knockdown or knockout of Lnc-408 in invasive BC cells clearly decreased LIMK1 levels, and ectopic Lnc-408 in MCF-7 cells increased LIMK1 expression to promote cell invasion. Lnc-408-mediated enhancement of LIMK1 plays a key role in cytoskeletal stability and promotes invadopodium formation in BC cells via p-cofilin/F-actin. In addition, the increased LIMK1 also facilitates the expression of MMP2, ITGB1, and COL1A1 by phosphorylating CREB. In conclusion, our findings reveal that Lnc-408 promotes BC invasion and metastasis via the Lnc-408/miR-654-5p/LIMK1 axis, highlighting a novel promising target for the diagnosis and treatment of BC.

## Introduction

Breast cancer (BC) is currently the most common malignancy among women globally, accounting for 30% of all female cancers [1]. In most cases, BC invasion and metastasis are the main cause of death. After the primary tumor cells spread to other locations in various ways, they form new lesions and eventually lead to death in most BC patients [2]. BC invasion and metastasis involve the processes of epithelial-mesenchymal transformation (EMT) and mesenchymal-epithelial transformation. EMT, an essential process during metastasis [3], enhances the ability of cancer cells to migrate, invade, and reshape the extracellular matrix (ECM), breaks the dormant state of cancer stem cells (CSCs), and triggers recurrence [4]. Research-based on EMT could provide inspiration to understand the invasion and metastasis of BC.

Long noncoding RNAs (lncRNAs) are RNA with a length greater than 200 nt and without protein-coding functions. LncRNAs are characterized by their abundant quantity, diversity, and mechanisms of action [5]. With the development of high-throughput RNA microarray and second-generation sequencing technologies, a large number of lncRNAs have been identified with their functions gradually revealed. Increasing evidence has shown that lncRNAs affect many cancer biological processes, including carcinogenesis, apoptosis, differentiation, proliferation, invasion, and metastasis [6]. Abnormal lncRNA expression is involved in regulating the expression of oncogenes or tumor suppressor genes, affecting the invasion and metastasis of BC [7–9]. LncRNAs have also been shown to play a role in EMT. For example, lncRNA-ANCR impedes TGF-β1-induced EMT by inhibiting the phosphorylation of RUNX2 [10]. In studies investigating the regulatory mechanisms of lncRNAs, researchers have proposed that lncRNAs work as “sponges” antagonizing the biological function of miRNAs by chelating miRNAs from their target mRNAs; these lncRNAs are known as competitive endogenous RNAs (ceRNAs) [11, 12]. For example, H19 was abnormally upregulated in BC and promoted BC cell invasion via the miR-152/DNMT1 axis, while overexpression of miR-152 or silencing of DNMT1 reversed this result [13]. Although BC researches remain at the forefront of cancer researches, studies on the relationship between lncRNAs and BC metastasis remain scarce.

In previous studies, we found that lncRNAs are involved in the regulation of EMT and related signaling pathways, as well as in metastasis, tumor cell metabolism, CSC enrichment, and maintenance of stemness in BC [14, 15]. Taking advantage of microarray technology, proteomics, and bioinformatics methods, we compared genes of CSCs with those of normal epithelioid cancer cells and found that lncRNAs (such as Hedgehog-specific lncRNA-Hh) affect CSC regulation in BC [16]. In this study, we applied an RNA microarray in BC cells undergoing EMT or BC tumor with lymph node metastasis compared with normal (control) BC cells or tumor without lymphatic metastasis. Based on this analysis, we identified some novel lncRNAs that exhibited significant expression changes. Among them, Lnc-408 showed a close association with poor prognosis in patients with BC. Lnc-408 could upregulate LIMK1 by serving as a sponge for miR-654-5p, which enhances the invasiveness and metastatic capacity of BC cells by activating p-cofilin/F-actin and p-CREB and downstream targets (MMP2/ITGB1/COL1A1). Our data suggest that Lnc-408 might be a new target for the diagnosis and treatment of breast cancer.

## Results

### High expression of Lnc-408 is associated with breast cancer progression

**Fig. 1:**
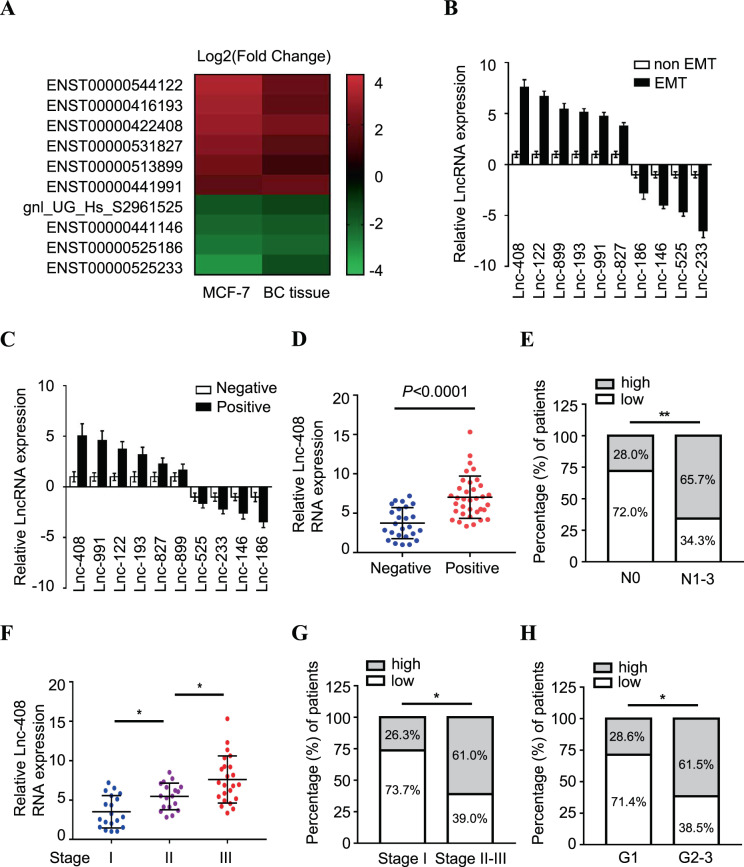
High expression of Lnc-408 is correlated with breast cancer progression. **A** The log2 (fold change) of the top ten differentially expressed lncRNAs in MCF-7/Twist versus MCF-7/Vector and BC tissue with (*N* = 3) versus without (*N* = 3) lymph node metastasis based on lncRNA microarray. **B**, **C** Ten randomly selected differentially expressed lncRNAs identified by lncRNA microarray were confirmed by qRT-PCR in MCF-7/Twist and MCF-7/Vector cells (**B**) or in BC tissue with (*N* = 3) and without (*N* = 3) lymph node metastasis (**C**). **D**, **F** qRT-PCR was used to detect Lnc-408 levels in patients with BC with (*N* = 35) or without (*N* = 25) lymph node metastasis (**D**) or BC tissues from different pathological stages (stage I (*N* = 19), stage II (*N* = 18), stage III (*N* = 23)) (**F**). **E**, **G** Percentages of tissues with low or high expression of Lnc-408 classified by N stage (**E**) or classified by TNM stage (**G**). **H** Percentages of tissues with low or high expression of Lnc-408 according to histological grade. (*P < 0.05, **P < 0.01, ***P < 0.001).

EMT is closely associated with malignant progression, including tumor cell invasion and metastasis [3], and lncRNAs have been reported to play multifunctional roles in tumor development [6]. To identify how lncRNAs influence breast cancer invasion and metastasis, an RNA microarray was carried out to scan aberrant lncRNAs associated with tumor invasion and metastasis using Twist-induced EMT cells (MCF-7/Twist) and corresponding epithelial cells (MCF-7/Vec), as well as three BC tissues with or without lymphatic metastasis. A set of dysregulated lncRNAs was identified in the Twist-induced mesenchymal tumor cells and BC tissues with lymphatic metastasis. The ten lncRNAs that underwent the highest increase or decrease (cut-off criteria of fold change >2.0 and *p* < 0.05) are shown in the heatmap (Fig. 1A). After confirmation by qRT-PCR, Lnc-408 was the lncRNA exhibiting the largest change in EMT-related BC cells (Fig. 1B, C, Supplementary Table 1) and was chosen for further research. To determine whether Lnc-408 had a potential effect on EMT features, we knocked down Lnc-408 in MCF7-Twist cells and examined EMT-related biomarker changes. Our data showed that loss of Lnc-408 could partially reverse the EMT phenotype, for example, by slightly increasing E-cadherin expression, decreasing N-cadherin and fibronectin expression, and correspondingly partially reducing cell motility induced by Twist1 (Fig. S1A–C). These results indicated that Lnc-408 is a potential target of Twist1 and is involved in tumor invasion and metastasis. To confirm the correlation of Lnc-408 with BC malignancy, we evaluated Lnc-408 levels in a set of BC cell lines (MCF-7, SKBr-3, T47D, MDA-MB-468, MDA-MB-231, BT-549, Hs578T) and tumor tissues from 60 patients with BC. The results showed that Lnc-408 was significantly upregulated EMT-related BC cells (Fig. S1D) and BC tissues with lymphatic metastasis compared with non-EMT BC cells and BC tissues without lymphatic metastasis (*P* < 0.0001) (Fig. 1D). The correlation between Lnc-408 expression and clinicopathological features of the patients with BC is listed in Supplementary Table 2 (low/high expression of Lnc-408 was defined by the median value of RNA relative expression). High expression of Lnc-408 was notably correlated with N stage (*P* = 0.0040) (Fig. 1E), TNM stage (*P* < 0.05) (Fig. 1F, G), and histological grade (*P* = 0.0149) (Fig. 1H). These data suggested that Lnc-408 participates in the development, and especially the invasion, of breast cancer.

### Lnc-408 promotes the migration and invasion of human breast cancer cells

**Fig. 2:**
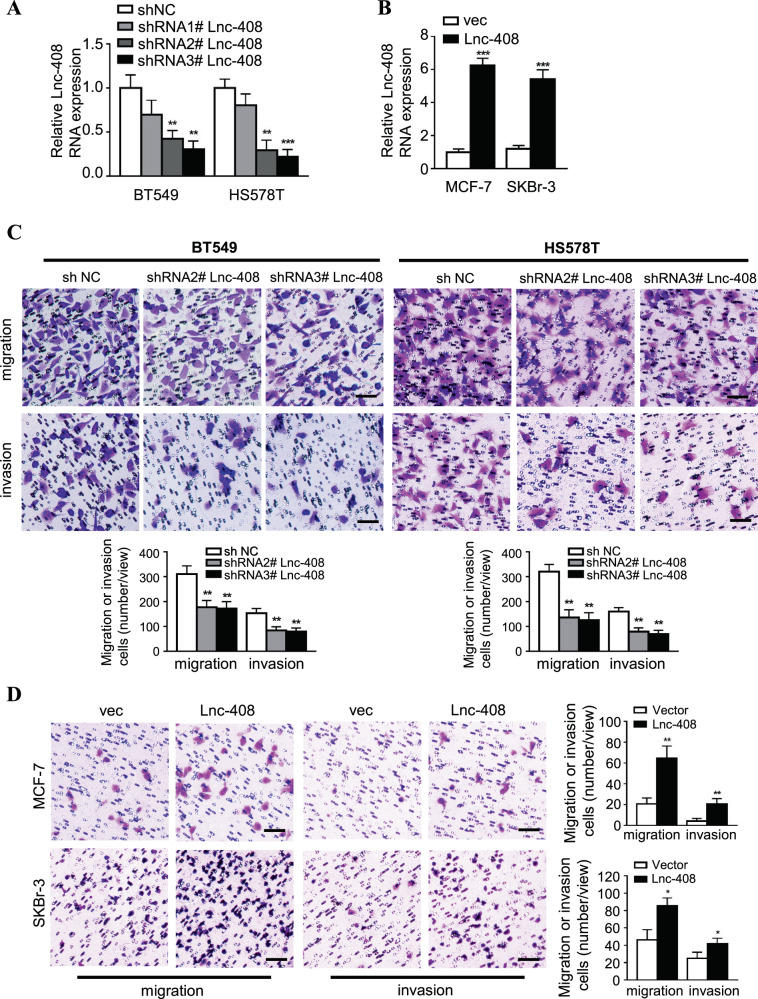
Lnc-408 promotes the migration and invasion of human breast cancer cells. **A** Expression of Lnc-408 in BT549 and Hs578T BC cells transfected with lentivirus-mediated shRNA against Lnc-408 (vs. shNC) detected by qRT-PCR. **B** Expression of Lnc-408 in the MCF-7 and SKBr-3 BC cell lines transfected with lentivirus-mediated Lnc-408 was determined by qRT-PCR. **C**, **D** Transwell assay to assess the migration and invasion abilities of BT549 and Hs578T cells with silenced Lnc-408 (**C**) or MCF-7 and SKBr-3 cells with ectopic Lnc-408 (**D**) (Scale bars, 100 μm). (*P < 0.05, **P < 0.01, ***P < 0.001).

As mentioned above, Lnc-408 was enhanced in strongly invasive BC cells (e.g., MDA-MB-231, BT-549, Hs578T, MDA-MB-468, and primary BC cell PL-BC-05) in contrast to weakly invasive BC cells (e.g., MCF-7, SKBr-3, T47D, and BC tissue without metastasis) (Fig. S1D). Thus, lentiviral vector-mediated Lnc-408 knockdown, Lnc-408 knockout, and Lnc-408-overexpressing cells were established (Figs. 2A, B, and S2A, B, Supplementary Table 3). Using transwell assays, we found that loss of Lnc-408 clearly impaired the migratory and invasive potentials of BT549, Hs578T, and PL-BC-05 cells (Figs. 2C and S2C), while ectopic Lnc-408 overexpression significantly promoted migration and invasion of MCF-7 and SKBr-3 cells (Fig. 2D), suggesting that Lnc-408 indeed exerts an oncogenic role in BC cells by promoting migration and invasion.

### LncRNA-408 regulates the expression of Lim domain kinase 1 (LIMK1)

**Fig. 3:**
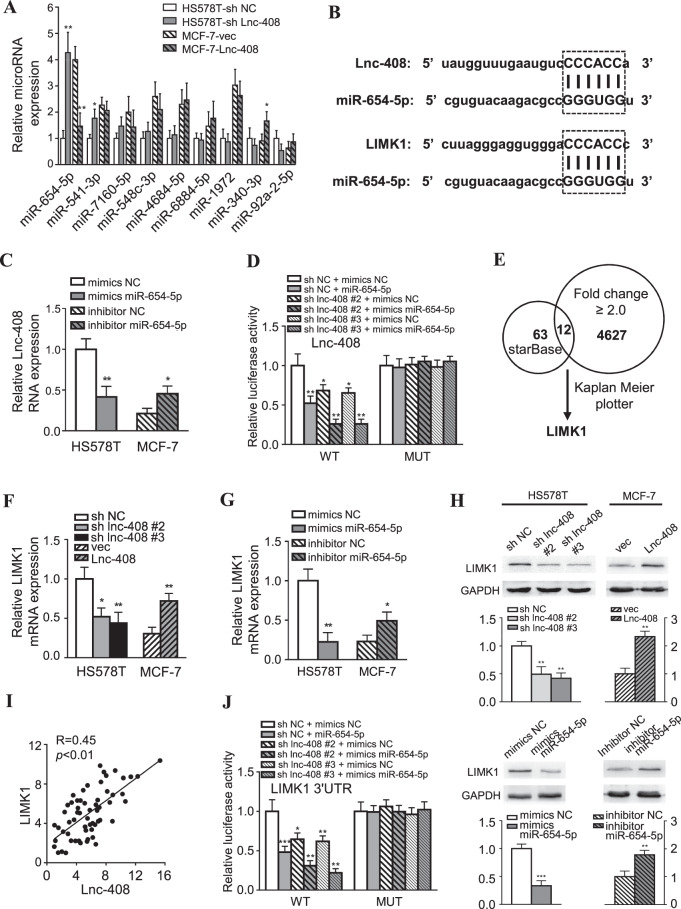
LncRNA-408 regulates the expression of LIMK1. **A** qRT-PCR was used to determine the target microRNA level in Lnc-408-silenced Hs578T cells and ectopic Lnc-408-overexpressing MCF-7 cells. **B** The miR-654-5p binding sites in Lnc-408 and LIMK1 mRNA predicted by MicroT-CDS. **C** qRT-PCR to detect Lnc-408 expression in Hs578T cells transfected with miR-654-5p mimics and MCF-7 cells treated with miR-654-5p inhibitor. **D** Hs578T/sh Lnc-408 and Hs578T/sh NC cells were transfected with Lnc-408-WT-luc or Lnc-408-MUT-luc along with miR-654-5p mimics or NC mimics, and the effect of miR-654-5p on Lnc-408 was determined by luciferase activity assay. **E** Schematic diagram showing the screening process for miR-654-5p target genes. **F**, **G** qRT-PCR was used to assess LIMK1 expression in Lnc-408-knockdown Hs578T cells and Lnc-408-overexpressing MCF-7 cells (**F**) and in Hs578T cells transfected with miR-654-5p mimics and MCF-7 cells treated with miR-654-5p inhibitor (**G**). **H** LIMK1 protein levels in the designed cells were evaluated by western blotting (band intensity was quantified using GAPDH as a normalizer). **I** Correlation between LIMK1 and Lnc-408 RNA levels in 60 clinical BC specimens. **J** Hs578T/sh Lnc-408 and Hs578T/sh NC cells were transfected with LIMK1-3′UTR-WT-luc or LIMK1-3′UTR-MUT-luc along with miR-654-5p mimics or NC mimics, and the effect of miR-654-5p on LIMK1 mRNA was determined by luciferase activity assay. (*P < 0.05, **P < 0.01, ***P < 0.001).

To explore the mechanism by which Lnc-408 impacts breast cancer cell migration and invasion, the subcellular distribution of Lnc-408 was assessed using nuclear and cytoplasmic extracts. Our data showed that Lnc-408 was mainly located in the cytoplasm of BC cells (Fig. S3A). Considering that lncRNAs can act as miRNA sponges in the cytoplasm, we predicted the potential miRNAs that might bind with Lnc-408 using the LncBase v.2 online databases. Fifteen miRNAs (Supplementary Table 4) were chosen based on a score greater than 0.8 and their levels were tested in Hs578T (shNC vs. sh Lnc-408) and MCF-7 (vector vs. ectopic Lnc-408) cells. The results showed that 9 miRNAs were relatively highly and stably expressed in the two groups of BC cells. Among them, miR-654-5p was significantly increased under silencing of Lnc-408, and notably declined upon Lnc-408 overexpression (Fig. 3A). According to the survival analysis from the TCGA BC database (*N* = 1262) based on Kaplan–Meier plots, a low level of miR-654-5p was significantly associated with poor prognosis in patients with BC (Fig. S3B). Moreover, miR-654-5p levels were negatively correlated with Lnc-408 levels in 60 BC samples (Fig. S3C). The potential interaction binding site was predicted using LncBase v.2 (Fig. 3B). In addition, ectopic miR-654-5p decreased Lnc-408 expression levels, and knockdown of miR-654-5p led to increased Lnc-408 in BC cells (Figs. 3C and S3D), indicating that miR-654-5p is a target miRNA of Lnc-408. The binding between Lnc-408 and miR-654-5p was further confirmed using a dual-luciferase reporter assay in Hs578T cells (Fig. 3D).

To identify miR-654-5p target genes, the StarBase v2.0 database was used to make predictions with the following filtering conditions: CLIP Data ≥2 (medium stringency), Degradome Data ≥2 (medium stringency), and program number ≥3. Sixty-three target genes were predicted (Fig. 3E). Based on the functional principle of ceRNA, the miR-654-5p target level should be positively correlated with the Lnc-408 level. These 63 genes intersected with 4627 upregulated mRNAs identified by RNA microarray in Twist-induced EMT cells, and 12 miR-654-5p targets were selected (Fig. 3E). Considering that high expression of Lnc-408 is closely related to invasive malignancy in BC, we analyzed the potential relationship between the expression of these targets in breast tumor tissues and patient survival using Kaplan–Meier plots from the TCGA database. We identified LIMK1 as the pertinent target of miR-654-5p (Fig. 3E). Predictions from LncBase v.2 indicated that Lnc-408 and LIMK1 could share the same miRNA response elements (MREs) of miR-654-5p (Fig. 3B), suggesting that Lnc-408 and miR-654-5p can regulate LIMK1 expression via a ceRNA mechanism. Indeed, loss of Lnc-408 significantly decreased LIMK1 levels in Hs578T and PL-BC-05 cells, and ectopic Lnc-408 increased LIMK1 expression in MCF-7 cells (Figs. 3F and S3D). miR-654-5p mimics reduced the expression of LIMK1 in Hs578T and PL-BC-05 cells, while a miR-654-5p inhibitor enhanced LIMK1 levels in MCF-7 cells (Figs. 3G, H and S3D). Furthermore, we found that LIMK1 expression was positively (*R* = 0.45) correlated with Lnc-408 levels (Fig. 3I); however, miR-654-5p negatively (*R* = 0.28) (Fig. S3E) correlated with Lnc-408 levels in our cohort of 60 patients with BC. As expected, the dual-luciferase reporter assay confirmed the binding between LIMK1 and miR-654-5p, which was analogous to the binding between Lnc-408 and miR-654-5p (Fig. 3J). Taken together, these data demonstrate that Lnc-408 serves as a sponge for miR-654-5p and impedes the function of miR-654-5p against its target gene LIMK1; that is, Lnc-408 can regulate LIMK1 via a ceRNA mechanism.

### LIMK1 enhances migration and invasion of BC cells

**Fig. 4:**
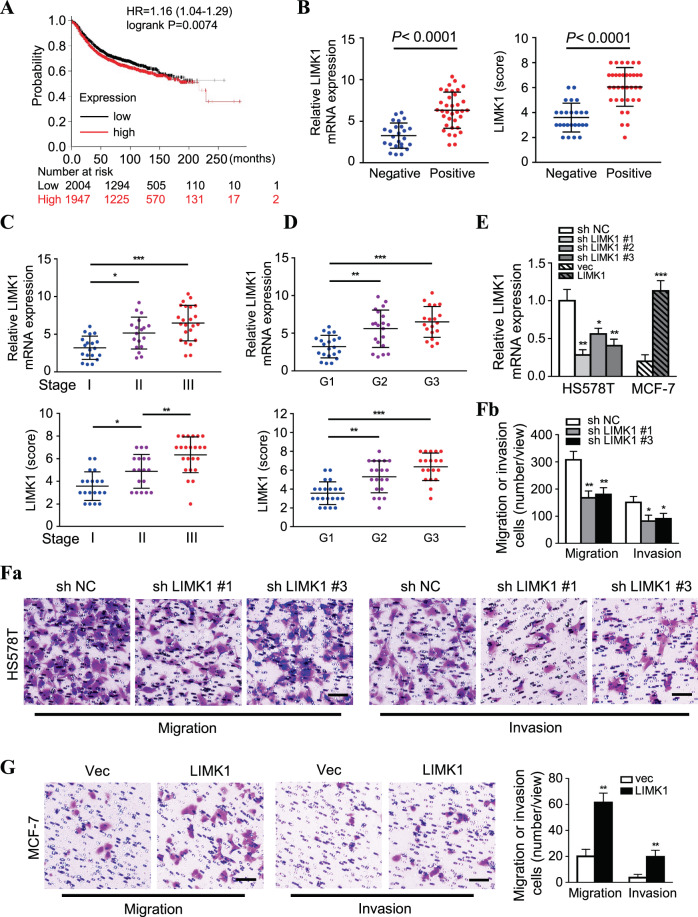
LIMK1 enhances migration and invasion of BC cells. **A** Survival analysis of patients with BC in the LIMK1 high and low expression groups (data source: kmplot breast cancer microarray database). **B** LIMK1 mRNA and protein levels in patients with BC with (*N* = 35) or without (*N* = 25) lymph node metastasis. **C**, **D** LIMK1 mRNA and protein levels in patients with different pathological stages(stage I (*N* = 19), stage II (*N* = 18), stage III (*N* = 23)) (**C**) or different pathological grades (G1 (*N* = 21), G2 (*N* = 20) and G3 (*N* = 19)) (**D**) of BC. **E** LIMK1 expression in Hs578T cells with silenced LIMK1 or MCF-7 cells with ectopic LIMK1 was determined by qRT-PCR. **F** (a, b) Transwell assay to assess migration and invasion of Hs578T cells with or without sh LIMK1 (Scale bars, 100 μm). **G** Invasion of MCF-7 cells transfected with ectopic LIMK1, assessed by Transwell assay (Scale bars, 100 μm). (*P < 0.05, **P < 0.01, ***P < 0.001).

Based on survival analysis using the TCGA BC database (*N* = 3951), we found that a high level of LIMK1 (Fig. 4A), but a low level of miR-654-5p (Fig. S3B), was closely correlated with poor prognosis in patients with BC. In our cohort, we further confirmed that LIMK1 expression (relative mRNA expression and IHC score) was significantly higher in BC tissues with lymphatic metastasis than in BC tissues without lymphatic metastasis (*P* < 0.0001) (Fig. 4B). Compared with levels in stage I or grade 1 BC tumor, LIMK1 mRNA levels were much higher in stage II (*P* = 0.0146) or grade 2 (*P* = 0.0013) and stage III (*P* < 0.0001) or grade 3 (*P* < 0.0001) BC tumor tissues (Fig. 4C, D). Similarly, the LIMK1 IHC scores gradually increased from stage I/grade 1 to stage II/grade 2 (*P* = 0.0228, *P* = 0.0011) and stage III/grade 3 (*P* < 0.0001, *P* < 0.0001) (Fig. 4C, D). LIMK1 IHC scores were also significantly higher in stage III compared with stage II (*P* = 0.0068) tumor (Fig. 4D), suggesting that LIMK1 is closely associated with the malignant progression of BC. Using lentivirus-mediated LIMK1 knockdown and overexpression in BC cells (Figs. 4E and S4A), we further confirmed that LIMK1 could enhance the migration and invasion of BC cells (Figs. 4F, G and S4B).

### LIMK1 can regulate p-cofilin/F-actin and p-CREB to fuel BC cell invasion

**Fig. 5:**
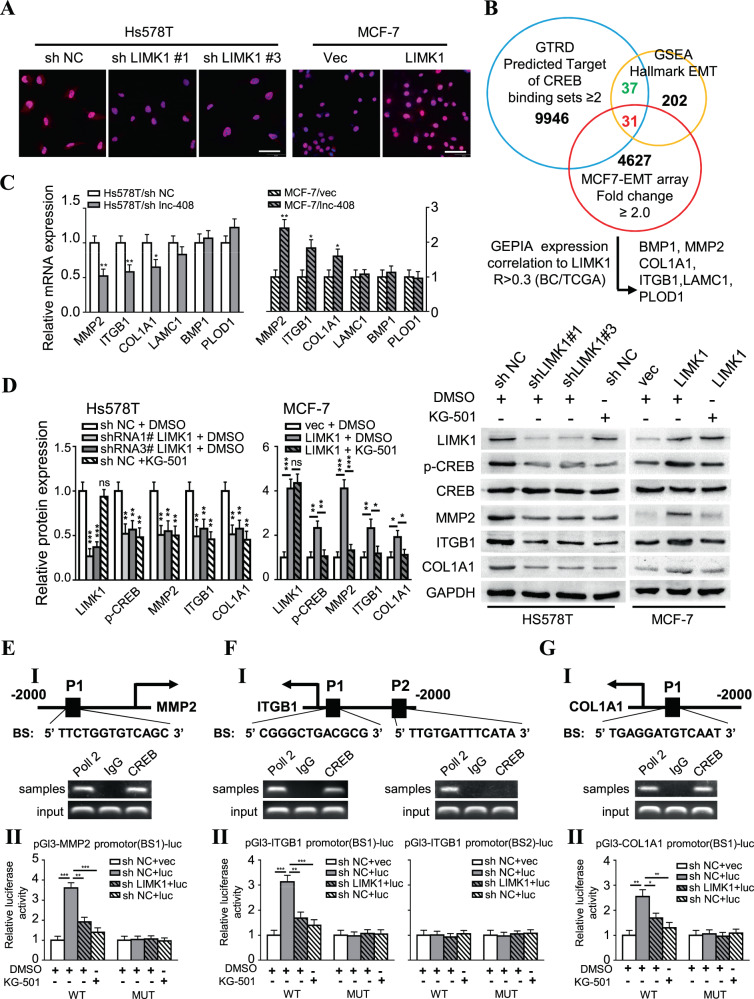
LIMK1 promotes BC cell invasion through p-CREB. **A** IF staining of p-CREB in LIMK1-silenced Hs578T cells or LIMK1-overexpressing MCF-7 cells (p-CREB was stained red, and nuclei were stained blue; scale bars, 50 μm). **B** The screening process for CREB target genes. **C** qRT-PCR was used to detect the mRNA expression of CREB target genes of interest in Lnc-408-silenced Hs578T cells and MCF-7 cells with ectopic Lnc-408. **D** Western blotting to evaluate the protein expression of LIMK1, p-CREB, CREB, MMP2, ITGB1, and COL1A1 in the designated BC cells (band intensity was quantified using GAPDH as a normalizer). **E**–**G**. (I) The predicted CREB binding sites in the promoters of MMP2, ITGB1, and COL1A1 based on JASPAR. (II) The relative luciferase activities of the target genes regulated by CREB in Hs578T cells (luc: pGI3-promptor-luc). (*P < 0.05, **P < 0.01, ***P < 0.001).

The LIMK1/cofilin/F-actin signaling axis has been reported to promote cell invasion in ovarian cancer [17] and non-small cell lung carcinomas [18]. High expression of matrix metalloproteinases (MMPs) is essential for cancer metastasis [19]. To explore whether LIMK1 could promote BC cell invasion by impacting cofilin/F-actin or MMP expression, we assessed the expression levels of the related key proteins. As expected, the expression of phosphorylated cofilin (p-cofilin) was significantly decreased in LIMK1 knockdown Hs578T and PL-BC-05 cells, and markedly increased in LIMK1-overexpressing MCF-7 cells (Fig. S4A). Correspondingly, cytoskeletal F-actin was reduced in LIMK1 knockdown Hs578T and PL-BC-05 cells, and increased in LIMK1-overexpressing MCF-7 cells, as verified by TRITC phalloidin staining, which showed clear changes in pseudopod and invadopodium formation in these cells (Fig. S4C). A previous report showed that LIMK1 could increase MMP2 expression in prostate cancer [20]. Thus, we queried the GEPIA 2 online analysis tool and calculated the correlation between LIMK1 and several recognized MMPs based on the TCGA BC database (*N* = 1085). MMP2 most closely correlated with LIMK1, with an *R*-value of 0.34 (Fig. S5A). There was a positive correlation between LIMK1 and Lnc-408 expression and MMP2 levels in our cohort of BC patients (Fig. S5B, C). In addition, immunohistochemistry (IHC) for LIMK1 and MMP2 in 60 BC samples showed a close correlation between LIMK1 and MMP2 based on their IHC scores (Fig. S5D); and representative cases are presented in Fig. S5E. On the other hand, a clear decrease of MMP2 was observed in LIMK1-knockdown Hs578T cells, and an increase of MMP2 in LIMK1-overexpressing MCF-7 cells (Fig. S5F), indicating that LIMK1 upregulates MMP2 expression. In addition to several classical substrates of LIMK1, CREB was reported to be phosphorylated by LIMK1 in H19-7 cells [21] and hippocampal tissue [22]. Employing bioinformatics, we identified a cAMP-response element (CRE) in the promoter region of the MMP2 gene, which might combine with CRE binding protein (CREB) to enhance the transcription of MMP2. Indeed, phosphorylated CREB (p-CREB) was notably reduced in LIMK1-knockdown Hs578T and PL-BC-05 cells and increased in ectopic LIMK1 MCF-7 cells (Figs. 5A and S5F, G), while total CREB levels were similar (Fig. S5F), suggesting that LIMK1 regulates MMP2 gene expression by phosphorylating CREB. To further search for additional CREB target genes that are potentially related to BC invasion and metastasis, we predicted 9946 CREB target genes with ≥2.0 binding sites using GTRD online tools. We then filtered them based on interactions with 202 hallmark EMT genes from GSEA and 4627 upregulated genes identified by RNA microarray in Twist-induced EMT cells. This process resulted in the selection of 31 potential target genes (Fig. 5B); and we identified six genes (BMP1, MMP2, COL1A1, ITGB1, LAMC1, and PLOD1) with an R-value >0.3 in the analysis of the correlation with LIMK1 expression from the TCGA BRCA data (*N* = 1085) provided by GEPIA (Fig. 5B). Finally, performed qRT-PCR and western blot analysis in Lnc-408-engineered BC cells, we confirmed that MMP2, ITGB1, and COL1A1 exhibited significant and substantial changes in Hs578T, MCF-7 cells (Fig. 5C, D), and primary BC tumor cells (Fig. S5H). Thus MMP2, ITGB1, and COL1A1 are the most likely CREB targets driving BC cell invasion and metastasis. We next predicted that there was a CREB binding site in the promoter of MMP2, ITGB1, and COL1A1 based on JASPAR; and this prediction was further proven by ChIP and luciferase reporter assays (Fig. 5E–G, Supplementary Table 5). The correlation between LIMK1 and ITGB1 or COL1A1 expression was similar to the correlation between MMP2 and LIMK1 expression (Fig. S6A, B). Clear decreases in ITGB1 and COL1A1 were also observed in LIMK1-knockdown Hs578T cells, and increases in LIMK1-overexpressing MCF-7 cells (Fig. 5D). To further confirm the function of p-CREB in regulating MMP2, ITGB1, and COL1A1, we treated BC cells with KG-501, a small molecule compound that can disrupt phosphor-CREB (Ser-133) binding to the KIX domain of CBP and results in a disrupted CREB-CBP complex, inhibiting CREB-target gene induction. KG-501 treatment led to significant decreases in MMP2, ITGB1, and COL1A1 at the protein and transcript levels in BC cells (Fig. 5D, E-II, F-II, G-II). These data show that LIMK1-mediated p-CREB effectively promotes the expression of MMP2, ITGB1, and COL1A1 in BC cells.

### Lnc-408 promotes BC cell invasion and metastasis through LIMK1

**Fig. 6:**
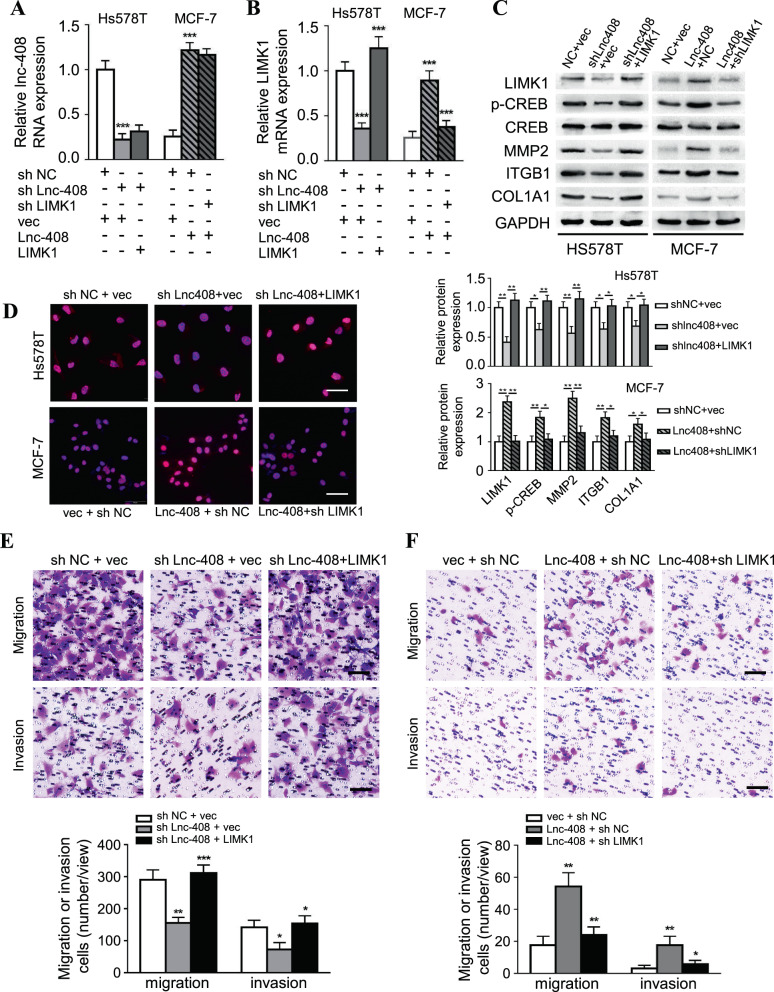
Lnc-408 promotes BC cell invasion via LIMK1. **A**, **B** qRT-PCR was used to detect Lnc-408 (**A**) or LIMK1 (**B**) expression in engineered Hs578T cells (Hs578T/sh Lnc-408, Hs578T/sh Lnc-408/LIMK1 and Hs578T/control) and engineered MCF-7 cells (MCF-7/Lnc-408, MCF-7/Lnc-408/sh LIMK1). **C** The protein expression levels of LIMK1, p-CREB, CREB, MMP2, ITGB1, and COL1A1 in the indicated BC cells were evaluated by western blotting (band intensity was quantified using GAPDH as a normalizer). **D** IF staining of p-CREB in the engineered Hs578T and MCF-7 cells described above, captured using fluorescence microscopy (p-CREB was stained red, and nuclei were stained blue with DAPI. Scale bars, 50 μm). **E**, **F** Transwell assays to assess the migration and invasion potential of the engineered Hs578T (**E**) and MCF-7 (**F**) cells described above (Scale bars, 100 μm). (*P < 0.05, **P < 0.01, ***P < 0.001).

To confirm the role of Lnc-408/LIMK1 signaling in tumor invasion, a set of engineered BC cells were established (Hs578T/Control, Hs578T/sh Lnc-408, and Hs578T/sh Lnc-408/LIMK1; PL-BC-05/Control, PL-BC-05/sh Lnc-408, and PL-BC-05/sh Lnc-408/LIMK1; PL-BC-05/Control, PL-BC-05/KO Lnc-408, and PL-BC-05/KO Lnc-408/LIMK1; MCF-7/Control, MCF-7/Lnc-408, MCF-7/Lnc-408/shLIMK1) (Figs. 6A, B and S7A). Loss of Lnc-408 led to decreased p-CREB, p-cofilin, MMP2, ITGB1, and COL1A1 protein levels, and ectopic LIMK1 restored p-CREB, p-cofilin, MMP2, ITGB1, and COL1A1 protein levels in Lnc-408-silenced Hs578T and PL-BC-05 cells; similarly, ectopic Lnc-408 in MCF-7 cells enhanced p-CREB, MMP2, ITGB1, and COL1A1 expression, and knockdown of LIMK1 attenuated the Lnc-408-induced increase in these proteins in MCF-7 cells (Figs. 6C, D and S7B–D). TRITC phalloidin staining showed that Lnc-408 knockdown impaired cytoskeletal F-actin and invadopodium formation, and ectopic LIMK1 corrected this impairment in Hs578T and PL-BC-05 cells (Fig. S8A). Conversely, ectopic Lnc-408 enhanced cytoskeletal F-actin and invadopodium formation, and loss of LIMK1 impaired ectopic Lnc-408-induced F-actin and invadopodium formation in MCF-7 cells (Fig. S8A). In addition, corresponding changes in cell migration and invasion were observed (Fig. 6E, F) in response to Lnc-408- and/or LIMK1-mediated cytoskeletal F-actin and invadopodium formation.

**Fig. 7:**
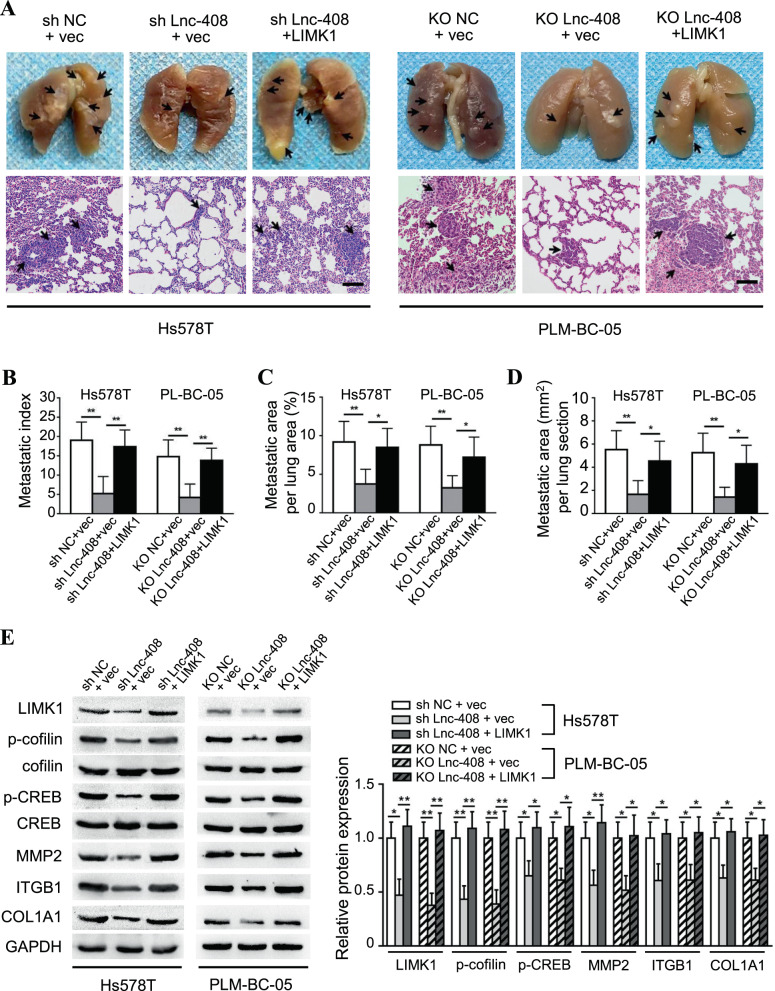
Lnc-408 promotes BC cell metastasis in vivo via LIMK1. **A** Representative images of surface metastases in whole lungs fixed in formalin (arrows show the metastatic nodule) and H&E staining of pulmonary metastatic foci (arrows show metastatic foci; scale bars, 100 μm). **B** Numbers of metastatic foci per lung. **C** Percentage of the metastatic area in the total lung area in each group of mice. **D** The average metastatic area (mm^2^) per lung section from five randomly selected lung sections of each mouse. **E** The expression levels of LIMK1, p-cofilin, cofilin, p-CREB, CREB, MMP2, ITGB1, and COL1A1 in tumor tissues were detected by western blotting. (*P < 0.05, **P < 0.01, ***P < 0.001).

**Fig. 8:**
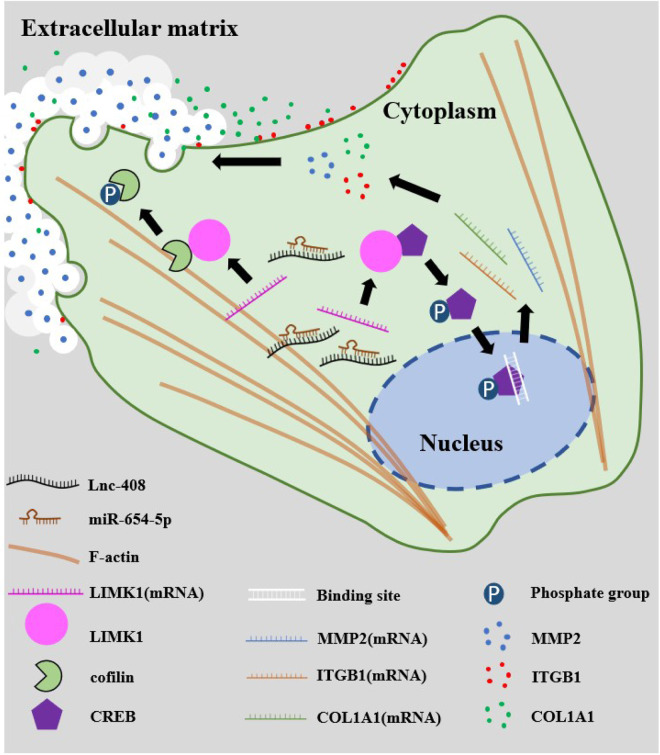
Schematic graph of Lnc-408 impacting BC invasion and metastasis. The enhanced Lnc-408 in BC cells serves as a sponge for miR-654-5p in the cytoplasm. Lnc-408-mediated inhibition of miR-654-5p impedes miR-654-5p targeting LIMK1 and results in the upregulation of LIMK1. The increased LIMK1 in BC maintains actin cytoskeletal organization via p-cofilin/F-actin. In addition, LIMK1 promotes the expression of MMP2, ITGB1, and COL1A1 by phosphorylating CREB, thus strengthening the invasive and metastatic potential of BC cells.

To expand our findings by evaluating the role of Lnc-408 and LIMK1 in metastasis, orthotopic xenografts were applied to nude mice by injecting engineered Hs578T cells and primary tumor cells (Hs578T/Control, Hs578T/sh Lnc-408, and Hs578T/sh Lnc-408/ectopic LIMK1; PL-BC-05/Control, PL-BC-05/KO Lnc-408, and PL-BC-05/KO Lnc-408/ectopic LIMK1) into the mammary fat pad. Notable metastatic nodules could be found in the lungs of tumor-burden mice injected with Hs578T/Control, PL-BC-05/Control, or Hs578T/sh Lnc-408/LIMK1 and PL-BC-05/KO Lnc-408/ectopic LIMK1, compared with mice injected with Hs578T/sh Lnc-408 and PL-BC-05/KO Lnc-408. The reduction in metastasis observed with silencing of Lnc-408 was reversed by ectopic LIMK1 overexpression (Fig. 7A–D), suggesting that Lnc-408-LIMK1 signaling plays an essential role in BC metastasis. The protein levels of LIMK1, p-cofilin, cofilin, p-CREB, CREB, MMP2, ITGB1, and COL1A1 in mouse tumor tissues were consistent with the metastatic potential of the tumor in mice (Fig. 7E). Taken together, these in vitro and in vivo data support the concept that Lnc-408 enhances BC invasion and metastasis by regulating LIMK1 (Fig. 8).

## Discussion

BC incidence is increasing globally and represents a serious health threat in women. The recurrence and metastasis of BC are a major challenge in clinical treatment and patient survival [23]. Here, we identified Lnc-408, a novel lncRNA that is highly expressed in BC cells undergoing EMT and in BC tumor with lymph node metastasis using RNA microarray analysis. Enhanced Lnc-408 levels are closely correlated with more advanced clinical stages and higher pathological grades in patients with BC. Our findings demonstrate that dysregulated lncRNAs, such as Lnc-408, play an essential role in BC metastasis and may function as potential novel targets for the diagnosis, prognosis, or even therapy in patients with BC.

Studies have shown that lncRNAs are related to cancer development, particularly during invasion and metastasis. The earliest known tumor-related lncRNAs, such as H19 which were found to promote tumor-related angiogenesis, inhibit tumor cell apoptosis, and increase the proliferation and hypoxia tolerance of tumor cells [24]. HOTAIR (HOX transcribed antisense RNA) was the first lncRNA found to be associated with tumor metastasis, and its upregulation mediates BC invasion and metastasis [25, 26]. An increasing number of lncRNAs have been found to be associated with BC invasion and metastasis in recent studies [27–29]. In our study, we confirmed that aberrant lncRNAs related to EMT are involved in the process of BC invasion and metastasis. Consistent with the methods used in other studies, confirmatory data from assays involving clinical specimens and BC cell models verified our assumption that Lnc-408 acts as a cancer promoter in the process of BC invasion and metastasis.

Among the different lncRNA regulatory mechanisms, ceRNAs have received the attention of researchers. The ceRNA hypothesis was first proposed by Pier Paolo Pandolfi, who suggested that RNA transcripts, including lncRNAs and mRNAs, compete for the same MRE to form a cross-talk network and regulate each other’s expression at the posttranscriptional level [30]. Accumulated evidence indicates that lncRNAs could serve as ceRNAs to impair the function of corresponding miRNAs and further regulate the expression of their target genes in tumor [11]. This interaction also occurs during cancer progression, especially during invasion and metastasis. For example, lncRNA HOTAIR is positively correlated with metastasis of BC [31]; HOTAIR acts as a sponge for miR-20a-5p and significantly influences the migration and invasion of tumor cells via the HOTAIR/miR-20a-5p/HMGA2 pathway [32]. The upregulation of LINC00461 enhances BC cell invasion by acting as a sponge for miR-30a-5p to regulate integrin [33], and the lncRNA BCRT1 promotes BC invasion by serving as a ceRNA for miR-1303 to relieve the suppressive effect of miR-1303 on its target, PTBP3 [34]. Here, we discovered that Lnc-408 and LIMK1 share the MRE of miR-654-5p. The negatively correlated expression pattern of miR-654-5p and Lnc-408 in BC tissues and engineered cells implies that Lnc-408 can act as a sponge for miR-654-5p in BC cells. Moreover, other reports have shown that miR-654-5p acts as a tumor suppressor in BC [35] and other cancers [36–38], which supports our finding that miR-654-5p antagonizes Lnc-408.

The Lnc-408 target LIMK1 was confirmed to functionally promote malignant progression in BC. LIMK1 is known to be a serine/threonine kinase that regulates actin polymerization by phosphorylation and inactivation of cofilin which is an actin depolymerizing factor (ADF). LIMK1 suppresses actin-severing activity and impacts actin cytoskeletal organization, including F-actin fiber arrangement [39] and invadopodium actin dynamics [40] by phosphorylating cofilin, which affects the process of invasion in different types of cancer [41, 42]. Consistent with previous studies, our study showed that knocking down Lnc-408 or LIMK1 impaired cytoskeletal F-actin and invadopodium formation in BC cells and that overexpressing LIMK1 in Lnc-408-silenced BC cells rescued these changes. However, beyond the changes of cancer cells themselves, the degradation of ECM is essential for cancer invasion, and LIMK1 has also been reported involved in this process [20]. MMPs, as a component of the ECM, are essential in tumor invasion and metastasis. Here, MMP2 levels were found to be closely correlated with LIMK1 expression. Our work revealed that Lnc-408-mediated upregulation of LIMK1 leads to CREB phosphorylation, which promotes the expression of MMP2 in BC cells, and in turn affects BC invasion and metastasis. In further exploration of the function of CREB, we confirmed that ITGB1 and COL1A1, which serve as components of the ECM and play important roles in BC cell invasion and metastasis, are also CREB target genes.

In conclusion, our study showed that Lnc-408 is highly expressed in BC cells and acts as a sponge for miR-654-5p in the cytoplasm. The inhibition of miR-654-5p by Lnc-408 blocks the binding between miR-654-5p and its target mRNA LIMK1, resulting in the upregulation of LIMK1. LIMK1 enhances the capacity of BC cells for invasion and metastasis of BC cells by maintaining actin cytoskeleton organization via p-cofilin/F-actin and enhancing the expression of MMP2, ITGB1, and COL1A1 via phosphorylated CREB.

## Materials and methods

### Human breast cancer tissue samples

Human breast tumor tissues were collected from patients with BC who underwent breast tumor resection at the First Affiliated Hospital of Chongqing Medical University, and the resected tissue specimens were immediately stored in liquid nitrogen. All patients involved provided formal informed consent. The research was approved by the Ethics Committee of Chongqing Medical University (Protocol No. 2018029A).

### Cell culture and reagents

Human breast cancer cells (MCF-7, SKBr-3, T47D, MDA-MB-468, MDA-MB-231, BT-549, Hs578T) were purchased from the American type culture collection (ATCC). The MCF-7/Twist and MCF-7/vector cells were constructed as described previously [16]. All BC cells were cultured in RPMI-1640 medium (Gibco, Grand Island, NY, USA) containing 10% FBS(Gibco). Cells were maintained in a humidified incubator at 37 °C with 5% CO_2_.

KG-501 was purchased from Selleck (Shanghai, China), and dimethyl sulfoxide (DMSO) was purchased from Sigma-Aldrich (St. Louis, MO, USA). Puromycin and G418 were purchased from Thermo Fisher Scientific (USA).

### Western blotting

Total protein was extracted from the indicated cells or tissues using RIPA lysis buffer (Beyotime, China), and the protein concentration was measured using a BCA protein assay kit (Beyotime, China). The protein was separated by 10% SDS-PAGE and subjected to western blotting, as described previously [43]. The primary antibodies used in western blotting were as follows: LIMK1(1:1000, ab81046, Abcam), p-cofilin (1:1000, #3313, CST), cofilin (1:1000, #5175, CST), p-CREB(Ser133) (1:1000, #9198, CST), CREB (1:1000, #9197, CST), MMP2(1:1000, ab97779, Abcam), ITGB1(1:1000, ab134179, Abcam) and COL1A1(1:1000, #72026, CST); GAPDH (1:1000, Boster, Wuhan, China) was used as a loading control. Horseradish peroxidase–conjugated anti-mouse or anti-rabbit IgG antibodies (1:3000, Boster, Wuhan, China) were used as secondary antibodies, and the protein expression levels were visualized using an enhanced chemiluminescence system (Bio-Rad, Hercules, EDAUSA). Band intensities from three biological experiments were quantified by densitometry using ImageJ software.

### Transwell cell migration and invasion assay

Cell migration and invasion abilities were measured using a Transwell assay as described previously [44]. Briefly, 2 × 10^4^ BC cells were suspended in a 200 μl culture medium (serum-free). For the invasion assay, the cells were seeded into the upper well of an 8-μm-pore Boyden chamber (Millipore, Darmstadt, Germany) coated with Matrigel (1:7.5, Corning BioCoat, Bedford, OH, USA). While for the migration assay, the cells were directly seeded into the upper well without coating. In addition, 500 μl culture medium containing 10% FBS was added to the lower well. BT549 and Hs578T cells were incubated at 37 °C and allowed to invade towards the lower chamber for 8 h, while MCF-7 and SKBr-3 cells for 24 h. After removing the residual cells from the upper surface of the chamber, the invaded cells were stained with 0.05% purple crystal. The migrated or invaded cells were counted under a microscope (Nikon, Japan) in five randomly selected views, and the mean value was calculated. The experiment was repeated at least three times.

### Luciferase reporter assay

To analyze the potential competition between Lnc-408 and LIMK1 mRNA for binding to miR-654-5p, luciferase (Luc) plasmids containing the sequences of Lnc-408 or LIMK1 3′-UTR with a wild-type (WT) or mutant (MUT) binding site for miR-654-5p were synthesized by Genecreat (Wuhan, China) and inserted into the pmirGLO-basic reporter plasmid to obtain the WT or mutant pmirGLO-lnc408-Luc and pmirGLO-LIMK1 3′-UTR-Luc constructs. The corresponding WT or MUT Luc plasmid and the control Luc plasmid, along with miR-654-5p mimics or control mimics, were transfected into Hs578T/shNC or Hs578T/sh Lnc-408 cells using Lipofectamine 2000 (Invitrogen, Carlsbad, CA, USA). After incubation for 30 h, cell lysates were collected and analyzed for firefly and Renilla luciferase activity using the Dual-Luciferase Reporter System (Promega, Madison, WI, USA).

To test whether CREB regulates its targets MMP2, ITGB1, or COL1A1 at the transcript level, the promoters of MMP2, ITGB1 or COL1A1 with WT or MUT binding sites for CREB were synthesized by Genecreat (Wuhan, China) and inserted into the pGL3-basic reporter plasmid to obtain MMP2, ITGB1, and COL1A1 WT or MUT luciferase constructs. The corresponding WT and MUT luciferase plasmids and control plasmids were transfected into Hs578T/sh NC or Hs578T/sh LIMK1 cells. Then, Hs578T/sh NC cells were treated with or without KG-501, and Hs578T/shLIMK1 cells were treated with DMSO and incubated for 30 h to collect cell lysates for luciferase activity assays. The experiment was repeated at least three times.

### Immunofluorescent staining (IF)

The indicated cells were seeded on coverslips in 24-well plates and fixed with 4% paraformaldehyde when cells were greater than 50% confluent. IF was conducted as previously described [45]. The specific primary antibody p-CREB(Ser133) (1:500, CST), Cy3-labeled secondary antibody (Sigma, USA), and DAPI for nuclear staining were used for IF.

### Orthotopic xenografts and metastasis assay

Animal experiments were performed with permission from the animal care ethics committees at Chongqing Medical University (Protocol No. 2018029B). The indicated engineered Hs578T cells (2 × 10^6^ cells/group) were suspended in 100 μl of PBS:Matrigel at a ratio of 1:1 and injected into the mammary fat pad of 4-week-old female nude mice (*n* = 5/group; animals were allocated randomly to each experimental group). The nude mice were euthanized on the 45th day after implantation to harvest tumor and lungs for further research. As described previously [46], lungs were fixed with 4% formalin, and visible metastatic foci on the lung surface were counted. Paraffin-embedded lung samples were serially sectioned into 5 μm thick sections and then used in H&E staining to check for metastatic loci in the lungs. The metastatic area and total lung section area were measured using ImageJ software.

### Statistical analysis

The statistical analyses were conducted using SPSS standard version 18.0 and GraphPad Prism 7.0. The data are presented as the mean ± SD from at least three independently repeated experiments (eliminate data if >mean + 3SD or <mean − 3SD). The difference between two groups was analyzed by Student’s *t*-test, and the differences among three or more groups were evaluated by one-way ANOVA and Tukey’s test for multiple comparisons. Chi-square tests were performed for the clinical data. Correlations between the expressions of different genes were analyzed using the Pearson correlation coefficient. Survival analysis and log-rank tests were conducted using the Kaplan–Meier method. Statistical significance was considered when the *p*-value was less than 0.05.

## Supplementary information

Supplementary Table 1

Supplementary Table 2

Supplementary Table 3

Supplementary Table 4

Supplementary Table 5

Supplemental Figures

Supplementary legends

Supplementary Methods and Materials

ethic permission
