# Supplementary material for: Long non-coding RNA Lnc-408 promotes invasion and metastasis of breast cancer cell by regulating LIMK1
Source: Oncogene. 2021 Jun 2;40(24):4198–213. doi: 10.1038/s41388-021-01845-y (PMC8211561; doi:10.1038/s41388-021-01845-y)
Supplement: Supplementary file 1 — Supplementary Table 1 [file 41388_2021_1845_MOESM1_ESM.doc]

**Supplementary Table 1. Primer sequences used for PCR**

| **LncRNA** | **Primers（5’ to 3’）** | |
| --- | --- | --- |
| ENST00000544122 | Forward | CAGTAGAGACAAATTATCCCC |
|  | Reverse | TTGCCACATAACAAATTGCT |
| ENST00000416193 | Forward | TTCCAATCCGGGCCTGACACC |
|  | Reverse | ACCTCCCGATGAGCCTACGA |
| ENST00000422408 | Forward | TTCCATTCAGAGACACGCTCA |
|  | Reverse | CAGTCCGAAGAAGACACCT |
| ENST00000531827 | Forward | GAAGGTGCCCCTAATATCACA |
|  | Reverse | CTCATTCTTTCACTTGCCAA |
| ENST00000513899 | Forward | ATTATTAGCACTTTGACCCCT |
|  | Reverse | TCTTCTTTGCTGCATTCCT |
| ENST00000441991 | Forward | TATCCCCTTTGAATCCATGC |
|  | Reverse | TTGCCTCTCTTTGTAGACCA |
| gnl_UG_Hs_S2961525 | Forward | AGCACATTTCCAGACAACGAA |
|  | Reverse | CTCCAGGGGTACAATCCATC |
| ENST00000441146 | Forward | CTGAGCAGCACACACTAGC |
|  | Reverse | TAATTCCTTGAGCCCAGTGACA |
| ENST00000525186 | Forward | GCAACAGCATGTATGGCATC |
|  | Reverse | AAAACACTGTCTTGAATTGCAC |
| ENST00000525233 | Forward | GCACCTACTGAAACCGGAA |
|  | Reverse | CATAAATCTACCGCGTTTGCT |
| LIMK1 | Forward | ACTGCGGGCACTGCTACTA |
|  | Reverse | GCTTGCCATGAGATGAGGCT |
| MMP2 | Forward | GATACCCCTTTGACGGTAAGGA |
|  | Reverse | CCTTCTCCCAAGGTCCATAGC |
| ITGB1 | Forward | CCTACTTCTGCACGATGTGATG |
|  | Reverse | CCTTTGCTACGGTTGGTTACATT |
| COL1A1 | Forward | GTGCGATGACGTGATCTGTGA |
|  | Reverse | CGGTGGTTTCTTGGTCGGT |
| β-Actin | Forward | TGACGTGGACATCCGCAAAG |
|  | Reverse | CTGGAAGGTGGACAGCGAGG |
| Lnc-408 KO confirmation | Forward | ATCTCGGCCTCTGAATAGCTG |
|  | Reverse | CCATAGCACCAGGATCGTT |
