# Supplementary material for: Long non-coding RNA Lnc-408 promotes invasion and metastasis of breast cancer cell by regulating LIMK1
Source: Oncogene. 2021 Jun 2;40(24):4198–213. doi: 10.1038/s41388-021-01845-y (PMC8211561; doi:10.1038/s41388-021-01845-y)
Supplement: Supplementary file 2 — Supplementary Table 2 [file 41388_2021_1845_MOESM2_ESM.doc]

**Supplementary Table 2. Correlation between lnc-408 expression and clinicopathological features in 60 BC patients**

| Characteristic |  | All cases | Lnc-408 | | Chi-square | *P* value |
| --- | --- | --- | --- | --- | --- | --- |
|  |  |  | low | high |  |  |
| All cases |  | 60 | 30 | 30 |  |  |
| Age | <50 | 25 | 15 | 10 | 1.7141 | 0.1904 |
|  | ≥50 | 35 | 15 | 20 |  |  |
| Menopausal | Premenopausal | 24 | 14 | 10 | 1.1111 | 0.2918 |
|  | Postmenopausal | 36 | 16 | 20 |  |  |
| T stage | T1 | 26 | 15 | 11 | 1.0861 | 0.2974 |
|  | T2/T3/T4 | 34 | 15 | 19 |  |  |
| N stage | N0 | 25 | 18 | 7 | 8.2971 | 0.0040** |
|  | N1/N2/N3 | 35 | 12 | 23 |  |  |
| TNM stage | I | 19 | 14 | 5 | 6.2391 | 0.0125* |
|  | II/III | 41 | 16 | 25 |  |  |
| Grade | 1 | 21 | 15 | 6 | 5.9341 | 0.0149* |
|  | 2/3 | 39 | 15 | 24 |  |  |

* *P*<0.05, ** *P*<0.01

**The detailed patient information**

| **Case NO.** | **Age** | **Menopausal** | **TNM stage** | **T** | **N** | **Grade** | **Lnc-408 level** |
| --- | --- | --- | --- | --- | --- | --- | --- |
| PH-BC-01 | <50 | Postmenopausal | stage I | 1 | 0 | 1 | low |
| PH-BC-02 | ≥50 | Postmenopausal | stage II | 2 | 1 | 2 | low |
| PH-BC-03 | <50 | Premenopausal | stage III | 2 | 2 | 2 | high |
| PH-BC-04 | ≥50 | Postmenopausal | stage III | 3 | 2 | 2 | high |
| PH-BC-05 | <50 | Premenopausal | stage I | 1 | 0 | 1 | low |
| PH-BC-06 | <50 | Postmenopausal | stage III | 2 | 2 | 3 | high |
| PH-BC-07 | ≥50 | Postmenopausal | stage III | 3 | 1 | 2 | low |
| PH-BC-08 | <50 | Premenopausal | stage II | 2 | 0 | 1 | low |
| PH-BC-09 | ≥50 | Postmenopausal | stage II | 2 | 1 | 3 | low |
| PH-BC-10 | ≥50 | Premenopausal | stage III | 2 | 2 | 3 | high |
| PH-BC-11 | ≥50 | Postmenopausal | stage III | 1 | 3 | 3 | high |
| PH-BC-12 | <50 | Premenopausal | stage I | 1 | 0 | 2 | low |
| PH-BC-13 | ≥50 | Postmenopausal | stage III | 3 | 3 | 2 | high |
| PH-BC-14 | <50 | Premenopausal | stage III | 3 | 1 | 2 | low |
| PH-BC-15 | ≥50 | Premenopausal | stage II | 2 | 1 | 2 | high |
| PH-BC-16 | ≥50 | Postmenopausal | stage III | 4 | 1 | 2 | low |
| PH-BC-17 | ≥50 | Postmenopausal | stage II | 2 | 0 | 1 | high |
| PH-BC-18 | <50 | Premenopausal | stage II | 2 | 0 | 1 | low |
| PH-BC-19 | <50 | Premenopausal | stage III | 1 | 2 | 3 | high |
| PH-BC-20 | ≥50 | Postmenopausal | stage III | 1 | 2 | 3 | high |
| PH-BC-21 | ≥50 | Postmenopausal | stage I | 1 | 0 | 2 | low |
| PH-BC-22 | ≥50 | Postmenopausal | stage III | 2 | 2 | 2 | high |
| PH-BC-23 | <50 | Premenopausal | stage II | 1 | 1 | 3 | low |
| PH-BC-24 | <50 | Postmenopausal | stage II | 2 | 0 | 2 | high |
| PH-BC-25 | ≥50 | Postmenopausal | stage III | 3 | 2 | 3 | high |
| PH-BC-26 | <50 | Premenopausal | stage I | 1 | 0 | 1 | low |
| PH-BC-27 | ≥50 | Postmenopausal | stage I | 1 | 0 | 1 | low |
| PH-BC-28 | ≥50 | Premenopausal | stage II | 3 | 0 | 1 | low |
| PH-BC-29 | <50 | Premenopausal | stage III | 2 | 3 | 3 | high |
| PH-BC-30 | ≥50 | Postmenopausal | stage II | 1 | 1 | 2 | high |
| PH-BC-31 | <50 | Premenopausal | stage I | 1 | 0 | 2 | low |
| PH-BC-32 | ≥50 | Postmenopausal | stage II | 2 | 1 | 2 | low |
| PH-BC-33 | <50 | Premenopausal | stage I | 1 | 0 | 1 | high |
| PH-BC-34 | <50 | Premenopausal | stage III | 2 | 2 | 3 | high |
| PH-BC-35 | ≥50 | Postmenopausal | stage I | 1 | 0 | 1 | low |
| PH-BC-36 | ≥50 | Postmenopausal | stage II | 1 | 1 | 3 | high |
| PH-BC-37 | <50 | Premenopausal | stage I | 1 | 0 | 1 | high |
| PH-BC-38 | ≥50 | Postmenopausal | stage III | 3 | 2 | 3 | high |
| PH-BC-39 | ≥50 | Postmenopausal | stage I | 1 | 0 | 1 | high |
| PH-BC-40 | <50 | Premenopausal | stage I | 1 | 0 | 1 | low |
| PH-BC-41 | ≥50 | Postmenopausal | stage III | 3 | 1 | 2 | low |
| PH-BC-42 | <50 | Postmenopausal | stage I | 1 | 0 | 1 | low |
| PH-BC-43 | ≥50 | Postmenopausal | stage III | 3 | 3 | 3 | high |
| PH-BC-44 | ≥50 | Postmenopausal | stage II | 2 | 1 | 3 | low |
| PH-BC-45 | <50 | Premenopausal | stage I | 1 | 0 | 1 | high |
| PH-BC-46 | ≥50 | Postmenopausal | stage II | 1 | 1 | 2 | high |
| PH-BC-47 | ≥50 | Postmenopausal | stage III | 3 | 2 | 3 | low |
| PH-BC-48 | ≥50 | Postmenopausal | stage III | 2 | 2 | 3 | low |
| PH-BC-49 | <50 | Premenopausal | stage II | 2 | 1 | 2 | high |
| PH-BC-50 | ≥50 | Postmenopausal | stage II | 2 | 1 | 2 | high |
| PH-BC-51 | ≥50 | Postmenopausal | stage I | 1 | 0 | 1 | high |
| PH-BC-52 | ≥50 | Postmenopausal | stage I | 1 | 0 | 1 | low |
| PH-BC-53 | <50 | Premenopausal | stage III | 2 | 2 | 3 | low |
| PH-BC-54 | <50 | Premenopausal | stage II | 2 | 0 | 1 | low |
| PH-BC-55 | ≥50 | Postmenopausal | stage III | 2 | 2 | 2 | high |
| PH-BC-56 | <50 | Premenopausal | stage I | 1 | 0 | 1 | low |
| PH-BC-57 | ≥50 | Postmenopausal | stage II | 2 | 1 | 3 | high |
| PH-BC-58 | <50 | Premenopausal | stage I | 1 | 0 | 1 | low |
| PH-BC-59 | ≥50 | Postmenopausal | stage III | 2 | 3 | 3 | high |
| PH-BC-60 | ≥50 | Postmenopausal | stage I | 1 | 0 | 1 | low |
