# Supplementary material for: Long non-coding RNA Lnc-408 promotes invasion and metastasis of breast cancer cell by regulating LIMK1
Source: Oncogene. 2021 Jun 2;40(24):4198–213. doi: 10.1038/s41388-021-01845-y (PMC8211561; doi:10.1038/s41388-021-01845-y)
Supplement: Supplementary file 3 — Supplementary Table 3 [file 41388_2021_1845_MOESM3_ESM.doc]

**Supplementary Table 3. RNA sequences used for targeting the indicated genes**

| RNA | sequence |
| --- | --- |
| sh NC | TTCTCCGAACGTGTCACGT |
| shRNA1# Lnc-408 | GAGACAGGGTTTCACCATA |
| shRNA2# Lnc-408 | GCCTAGATATTCATTTCTA |
| shRNA3# Lnc-408 | GAACGTATTCCCAAAGAAA |
| shRNA1# LIMK1 | GGGTGCTCTACAAGGACAA |
| shRNA2# LIMK1  shRNA3# LIMK1 | GTCTCATGGTGGACGAGAA  GGCAGCTCTTCTGCAAGAA |
| sgRNA1# Lnc-408 forward | TCGCCTAGGCTGGAGTGCAG |
| sgRNA2# Lnc-408 forward | TTTTGGGGTTTTTTTTGAGA |
| sgRNA1# Lnc-408 reverse | CTTTTTCTGACCCTAGGAGA |
| sgRNA2# Lnc-408 reverse | TGGAAAGTGTGGTCCTCCAG |
