# Supplementary material for: Long non-coding RNA Lnc-408 promotes invasion and metastasis of breast cancer cell by regulating LIMK1
Source: Oncogene. 2021 Jun 2;40(24):4198–213. doi: 10.1038/s41388-021-01845-y (PMC8211561; doi:10.1038/s41388-021-01845-y)
Supplement: Supplementary file 4 — Supplementary Table 4 [file 41388_2021_1845_MOESM4_ESM.doc]

**Supplementary Table 4. Primer sequences for miRNA**

| **miRNA** | **Primers sequence（5’ to 3’）** | |
| --- | --- | --- |
| stem-loop：GTCGTATCCAGTGCAGGGTCCGAGGTATTCGCACTGGATACGAC | | |
| Reverse：AGTGCAGGGTCCGAGGTATT | | |
| miR-92a-2-5p | sequence behind stem-loop | GTAATG |
|  | Forward | CGGGGTGGGGATTTGTTG |
| miR-6716-5p | sequence behind stem-loop | GGCCCT |
|  | Forward | CGTCTGGGAATGGGGGTA |
| miR-7110-3p | sequence behind stem-loop | CTGCAG |
|  | Forward | CGCGTCTCTCTCCCACTTCC |
| miR-541-3p | sequence behind stem-loop | AGTCCA |
|  | Forward | GCGTGGTGGGCACAGAATC |
| miR-4684-5p | sequence behind stem-loop | TATGTT |
|  | Forward | CGCGCTCTCTACTGACTTGC |
| miR-6884-5p | sequence behind stem-loop | CAACAT |
|  | Forward | CGCGAGAGGCTGAGAAGGTG |
| miR-548c-3p | sequence behind stem-loop | GCAAAA |
|  | Forward | GCGCGCAAAAATCTCAATTAC |
| miR-340-3p | sequence behind stem-loop | GCTATA |
|  | Forward | GCGCGTCCGTCTCAGTTACTT |
| miR-654-5p | sequence behind stem-loop | GCACAT |
|  | Forward | TGGTGGGCCGCAGAAC |
| miR-7160-5p | sequence behind stem-loop | GGCACA |
|  | Forward | CGTGCTGAGGTCCGGGC |
| miR-4697-3p | sequence behind stem-loop | ACCAAG |
|  | Forward | CGTGTCAGTGACTCCTGCCC |
| miR-4677-3p | sequence behind stem-loop | AGTAGT |
|  | Forward | CGCGTCTGTGAGACCAAAGA |
| miR-6810-5p | sequence behind stem-loop | GCCATG |
|  | Forward | CGATGGGGACAGGGATCAG |
| miR-1972 | sequence behind stem-loop | TGAGCC |
|  | Forward | CGTCAGGCCAGGCACAGT |
| miR-1273g-3p | sequence behind stem-loop | CTCAGG |
|  | Forward | CGCGACCACTGCACTCCAG |
| miR-6769b-5p | sequence behind stem-loop | GCACTT |
|  | Forward | TGGTGGGTGGGGAGGAG |
| miR-519d-3p | sequence behind stem-loop | CACTCT |
|  | Forward | CGCAAAGTGCCTCCCTTT |
| miR-106b-5p | sequence behind stem-loop | ATCTGC |
|  | Forward | CGTAAAGTGCTGACAGT |
| miR-20a-5p | sequence behind stem-loop | CTACCT |
|  | Forward | CGTAAAGTGCTTATAGTGC |
| miR-106a-5p | sequence behind stem-loop | CTACCT |
|  | Forward | CGAAAAGTGCTTACAGTGC |
| miR-526b-3p | sequence behind stem-loop | GCCTCT |
|  | Forward | CGAAAGTGCTTCCTTTT |
| miR-328-3p | sequence behind stem-loop | ACGGAA |
|  | Forward | CGTCTGGGAATGGGGGTA |
| miR-7162-5p | sequence behind stem-loop | CAGCTG |
|  | Forward | CGCGTGCTTCCTTTCT |
| U6 | sequence behind stem-loop | AAAATA |
|  | Forward | AGAGAAGATTAGCATGGCCCCTG |
