# Supplementary material for: Long non-coding RNA Lnc-408 promotes invasion and metastasis of breast cancer cell by regulating LIMK1
Source: Oncogene. 2021 Jun 2;40(24):4198–213. doi: 10.1038/s41388-021-01845-y (PMC8211561; doi:10.1038/s41388-021-01845-y)
Supplement: Supplementary file 5 — Supplementary Table 5 [file 41388_2021_1845_MOESM5_ESM.doc]

**Supplementary Table 5. Primer sequences for amplification of the indicated promoter by PCR**

| **Gene** | **Position** | **Promoter Primers（5’ to 3’）** | |
| --- | --- | --- | --- |
| MMP2 | -1591 ~ -1579 | Forward | ACCCTAAGGTAAGTGATGA |
|  |  | Reverse | GGAAATTTAGCCAACATGA |
| ITGB1 | -201 ~ -184 | Forward | CGCGCAGCCGGTTCCTTTGTCC |
|  |  | Reverse | GCCTCCTCTGCGCGTCTGATCCC |
| ITGB1 | -1860 ~ -1848 | Forward | ATTTCCTGTTTAGCACGTA |
|  |  | Reverse | GCACTTGACTAAACTGATGT |
| COL1A1 | -674 ~ -662 | Forward | GAGGCCACCTAGTCATGTTT |
|  |  | Reverse | GGGGAACAATTTACAGATTCCTT |
| ENSG00000228274 | -1636 ~ -1624 | Forward | GAGCCACACAGCAGTAGATCA |
|  |  | Reverse | GCACTGTTCACAAGGGTTT |
