# Supplementary figures and images for: Long non-coding RNA Lnc-408 promotes invasion and metastasis of breast cancer cell by regulating LIMK1
Source: Oncogene. 2021 Jun 2;40(24):4198–213. doi: 10.1038/s41388-021-01845-y (PMC8211561; doi:10.1038/s41388-021-01845-y)

**Fig S1**

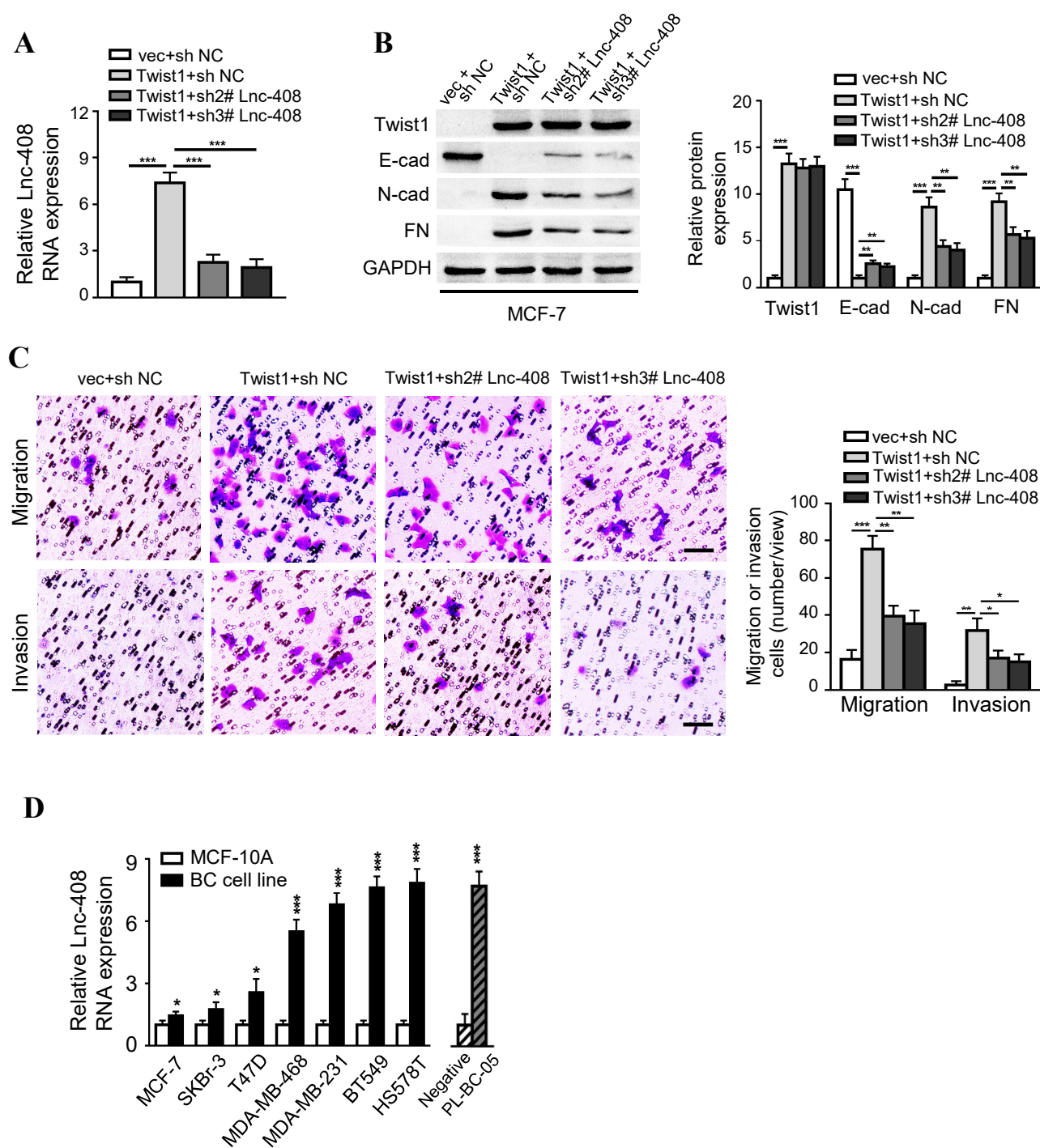

Fig S2

A

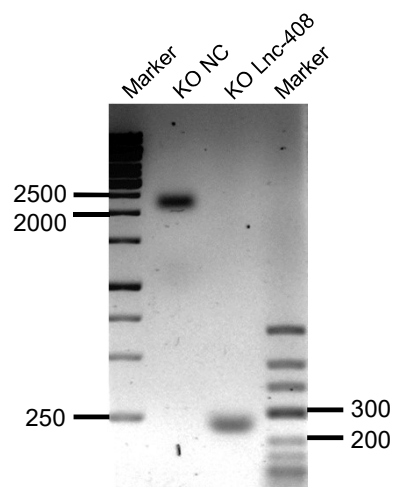

B

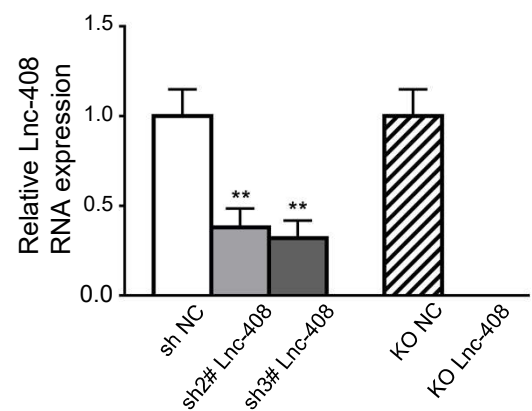

C

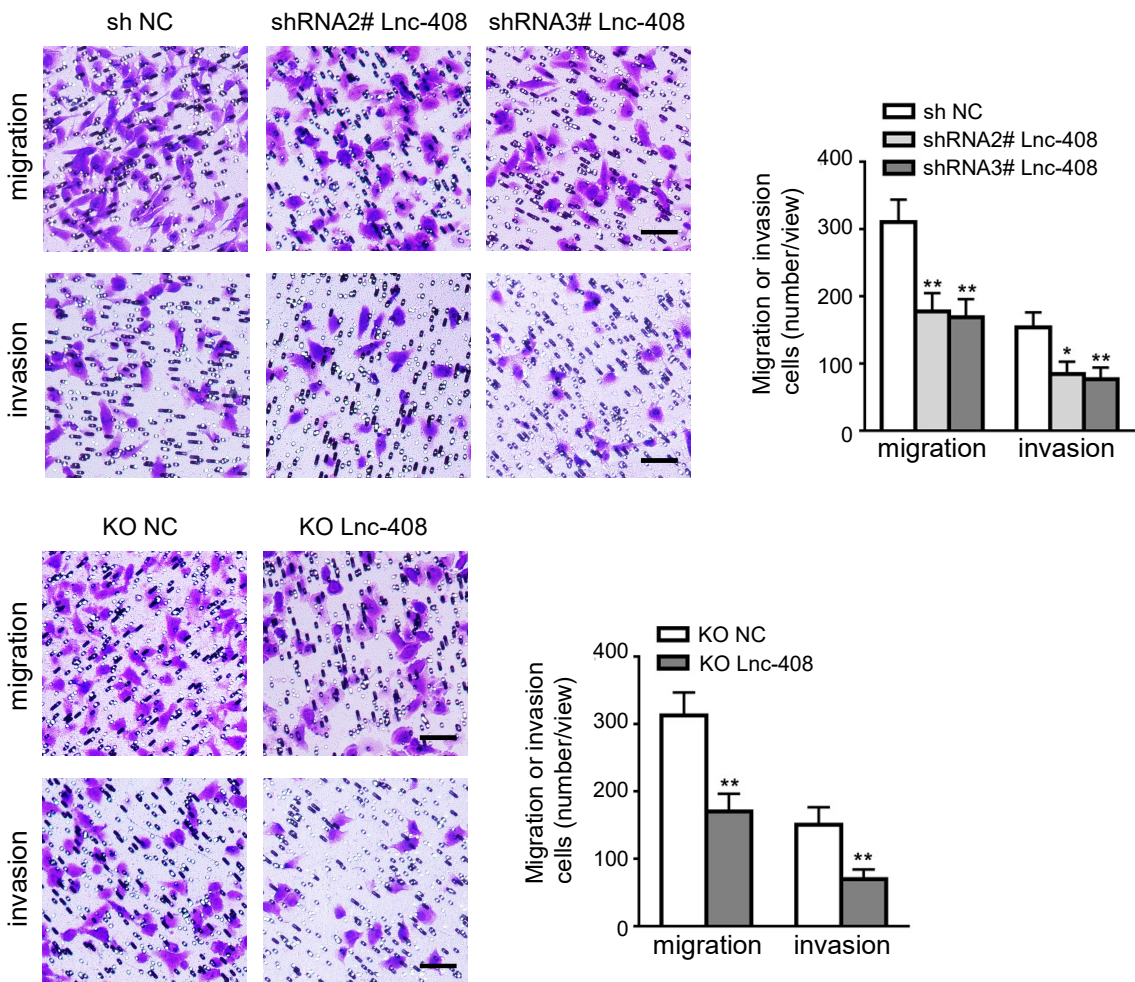

**Fig S3**

**A**

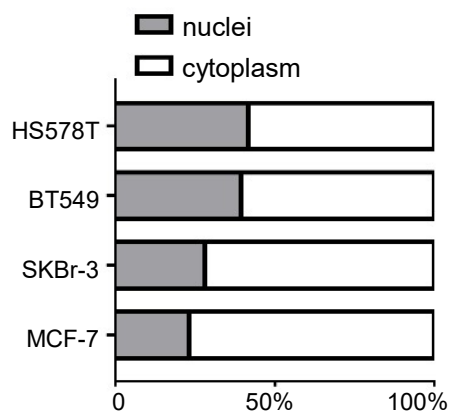

**B**

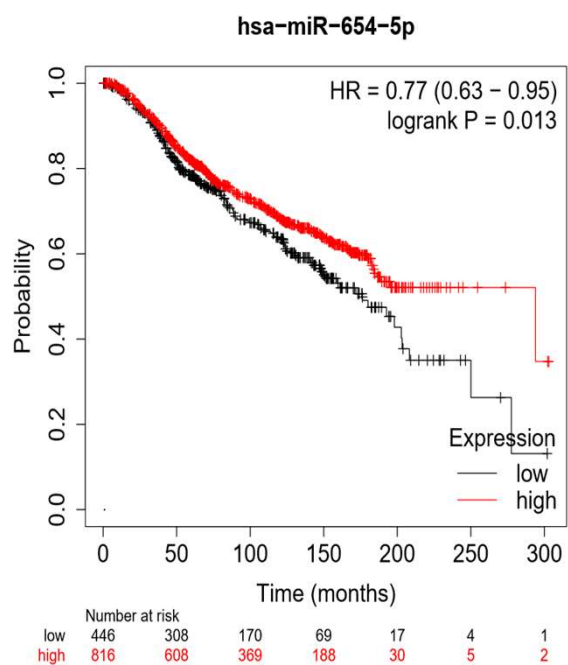

**C**

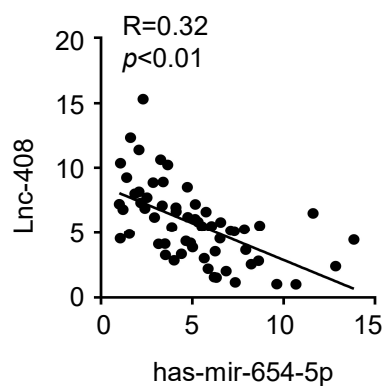

**D**

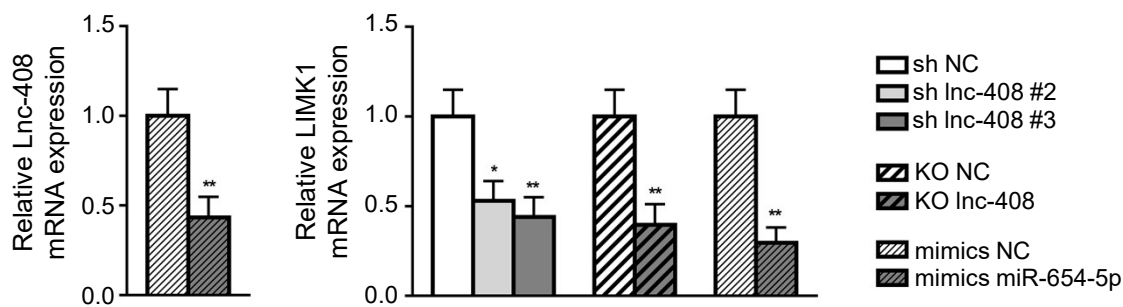

**E**

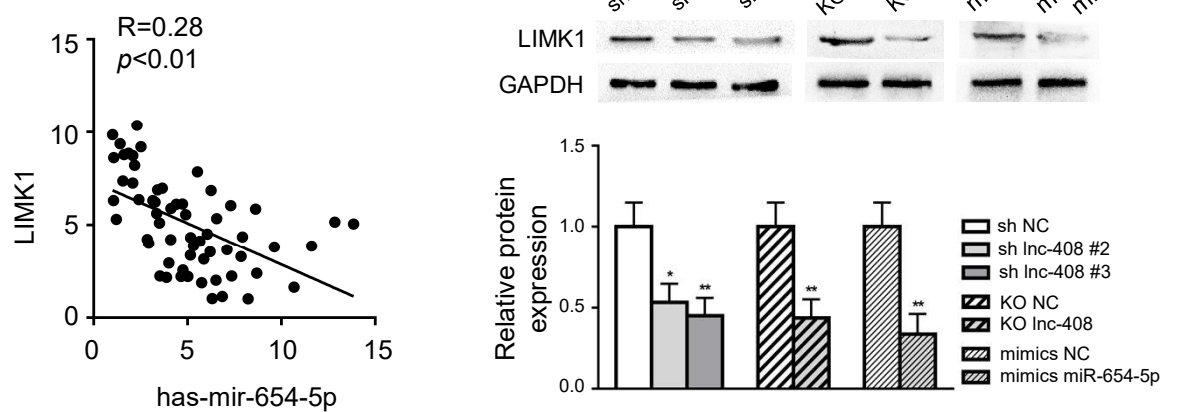

Fig S4

A

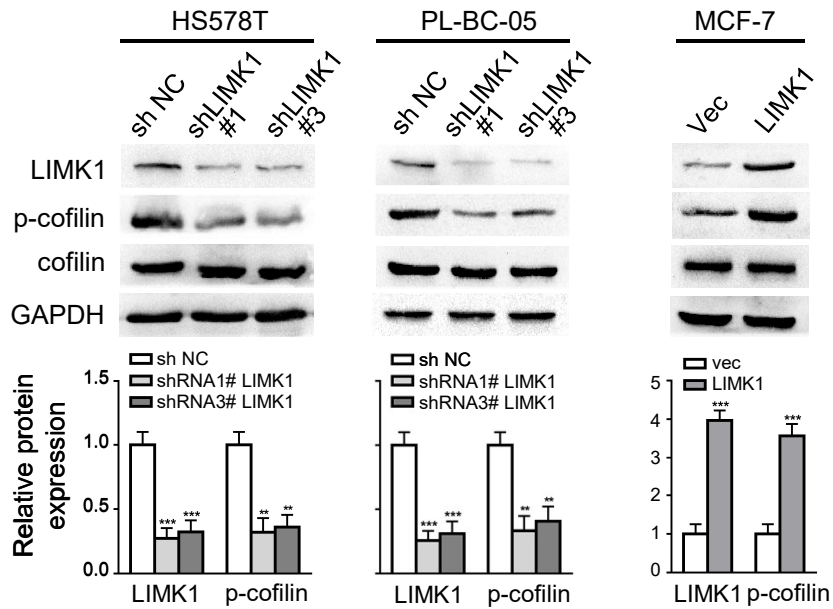

B

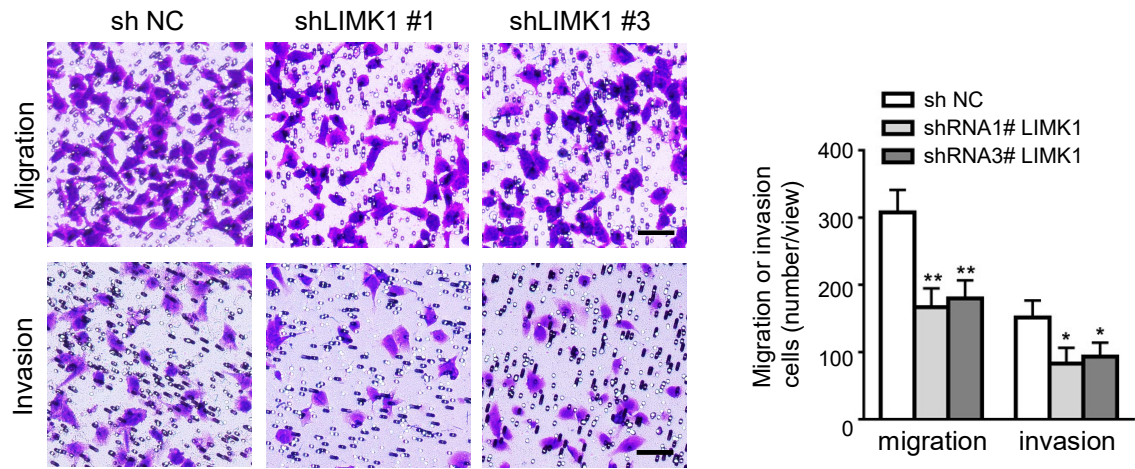

C

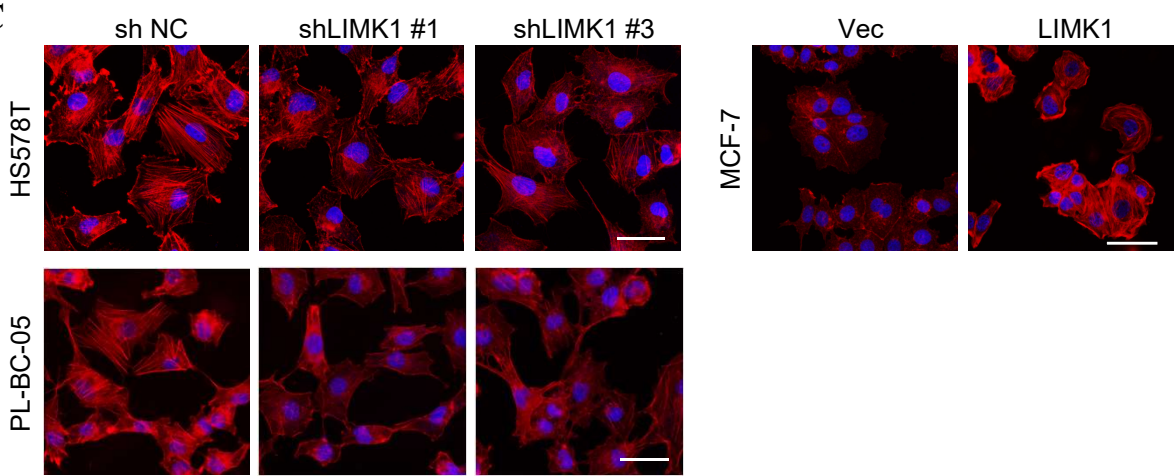

**Fig S5**

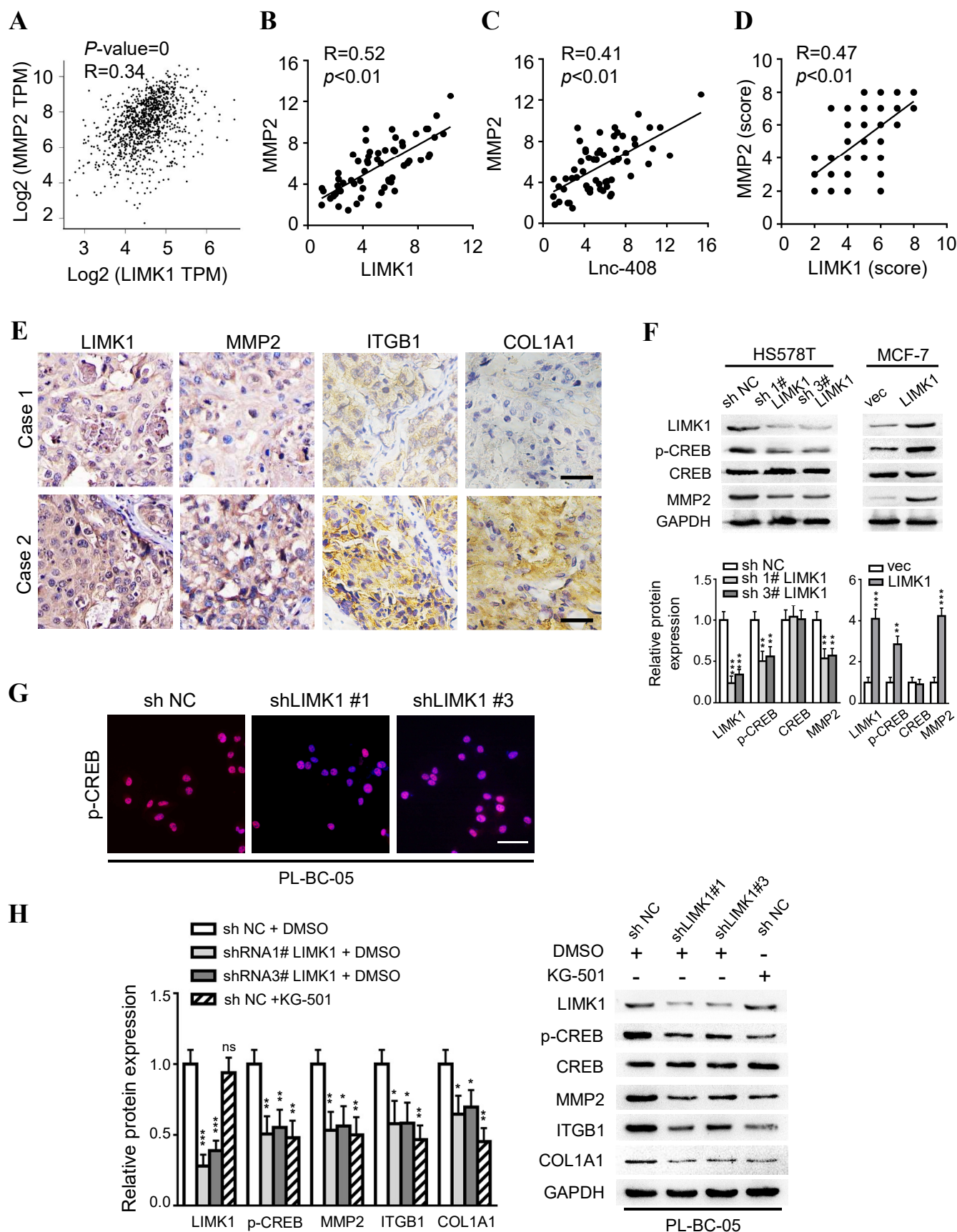

**Fig S6**

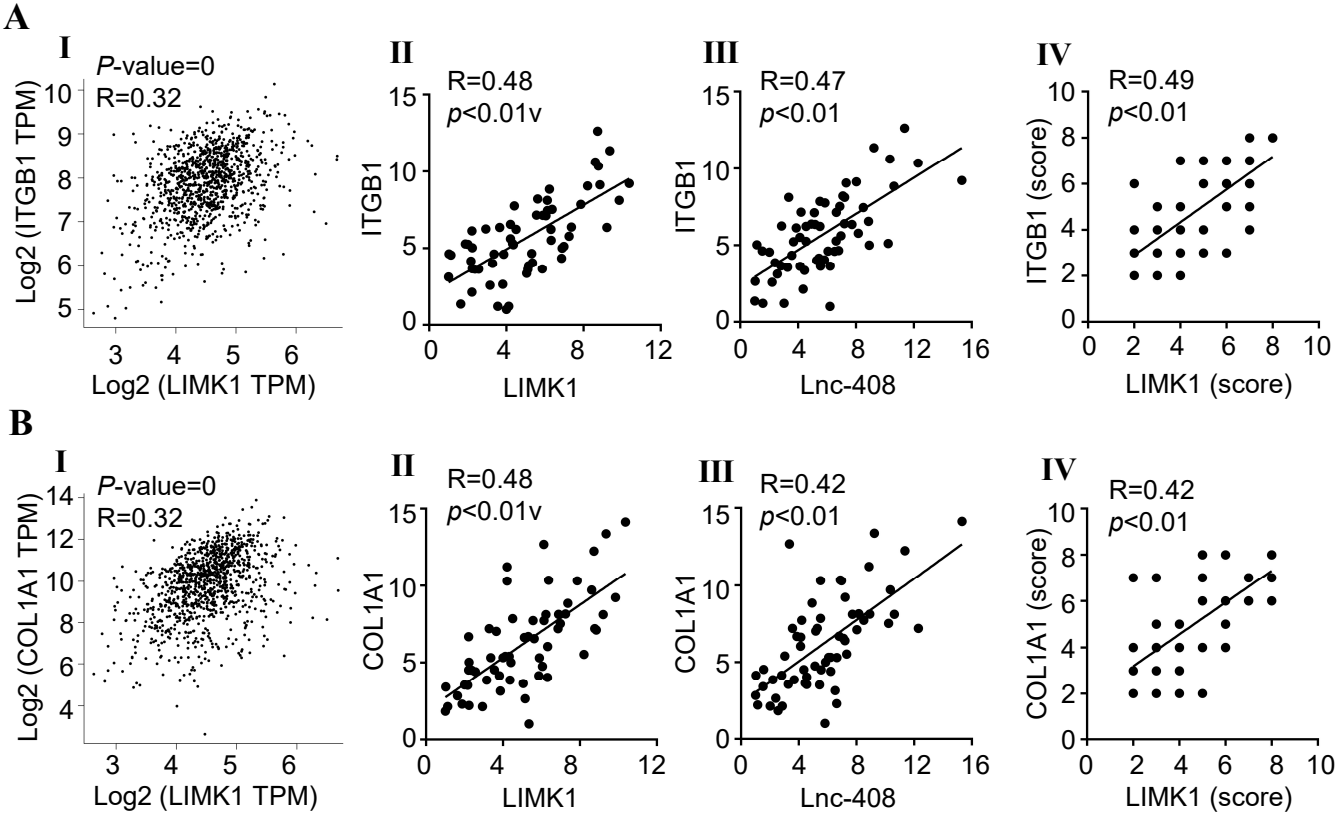

**Fig S7**

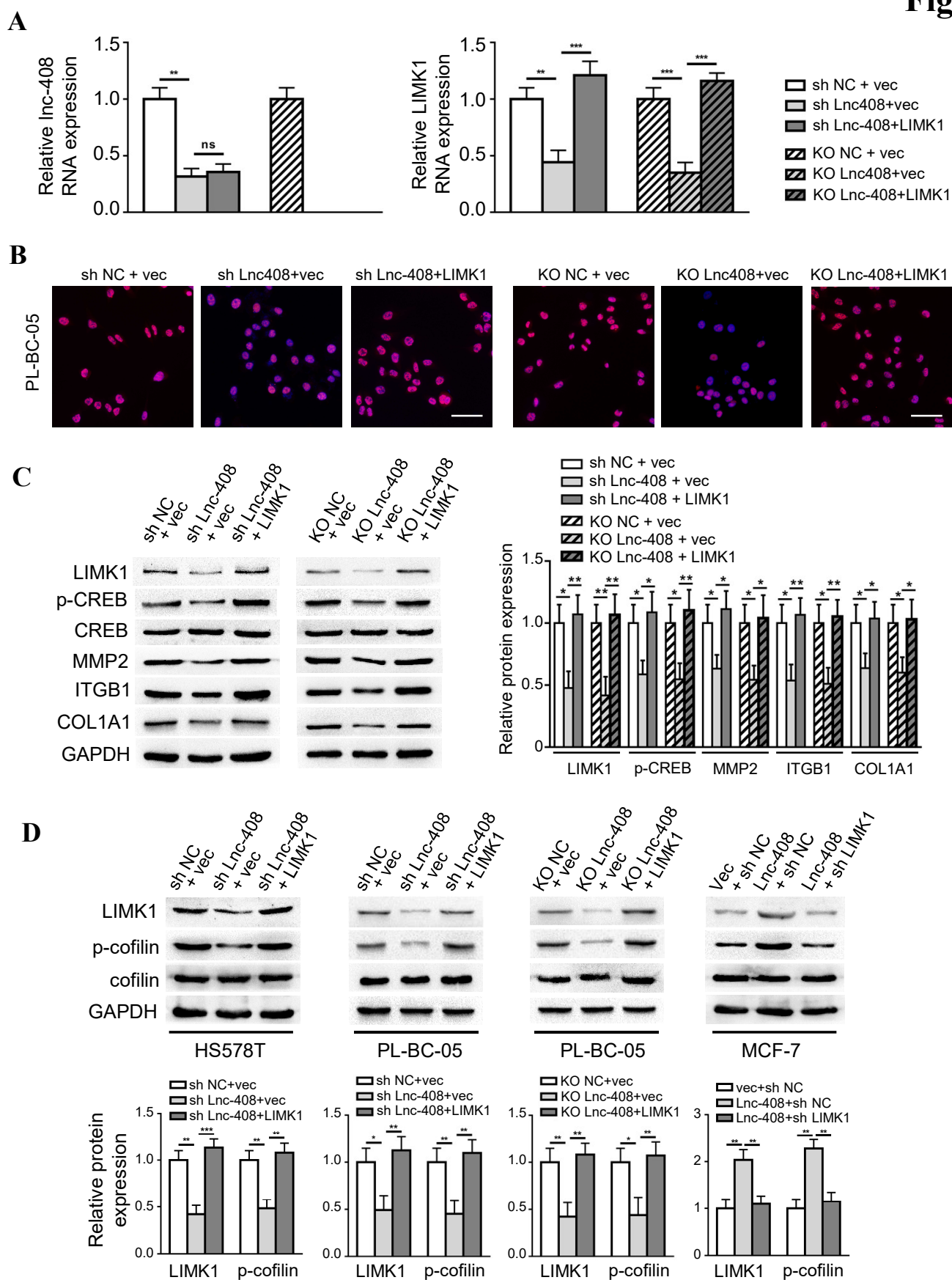

**Fig S8**

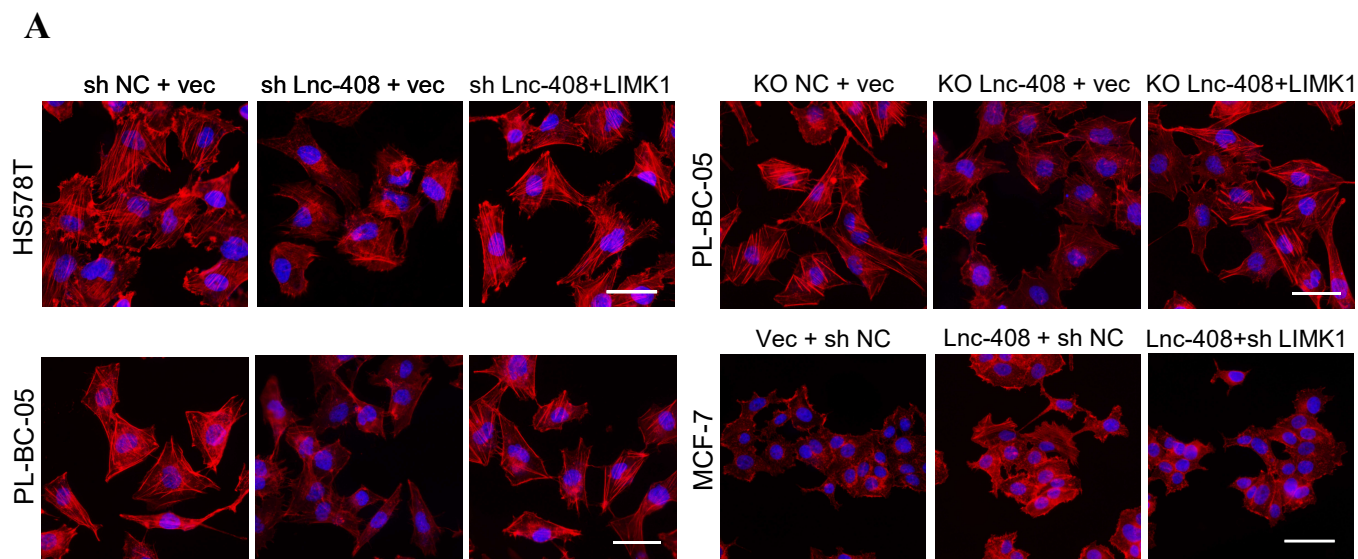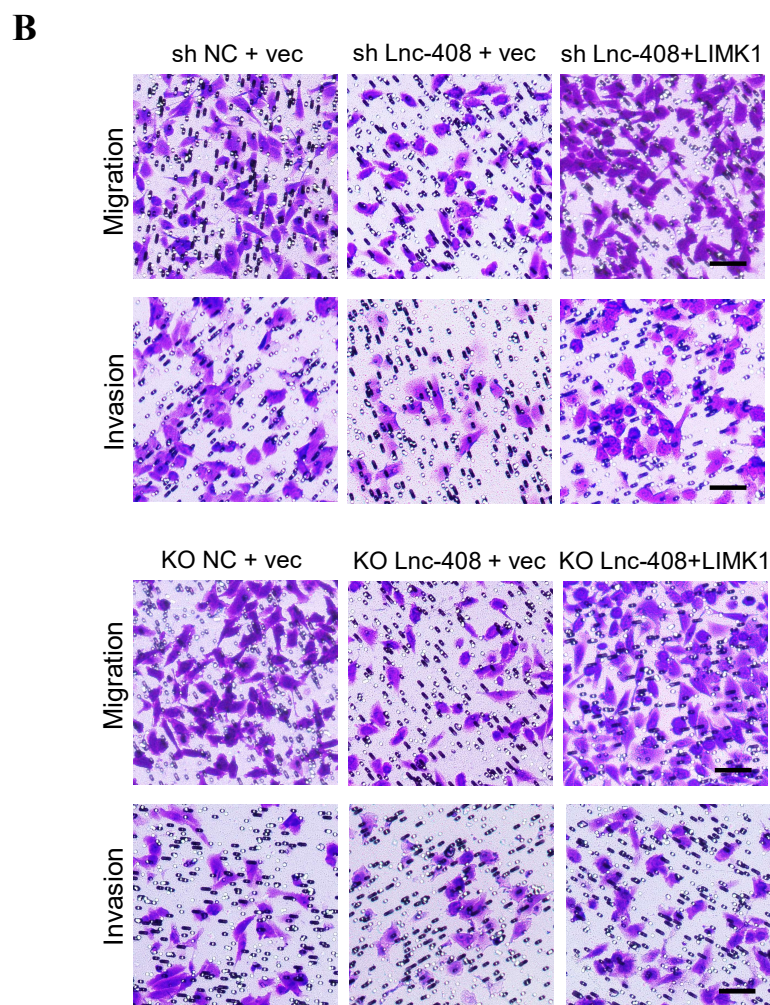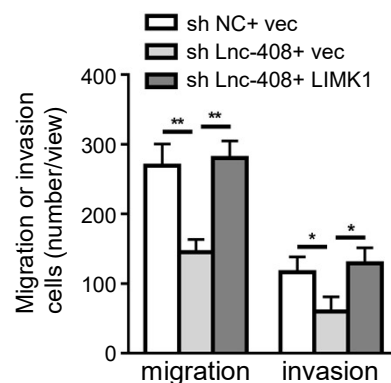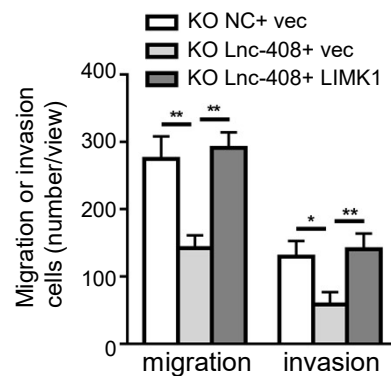

Supplement: Supplementary file 6 — Supplemental Figures [file 41388_2021_1845_MOESM6_ESM.pdf]
