# Supplementary material for: Long non-coding RNA Lnc-408 promotes invasion and metastasis of breast cancer cell by regulating LIMK1
Source: Oncogene. 2021 Jun 2;40(24):4198–213. doi: 10.1038/s41388-021-01845-y (PMC8211561; doi:10.1038/s41388-021-01845-y)
Supplement: Supplementary file 7 — Supplementary legends [file 41388_2021_1845_MOESM7_ESM.docx]

**Supplementary Figure Legends**

**Supplemental Figure 1. Lnc-408 suppression reversed EMT and the enhanced invasive phenotype caused by Twist1 overexpression in MCF-7 cells**

**A**. Lnc-408 levels were determined by qRT-PCR in the indicated MCF-7 cells. **B**. The protein levels of Twist1, E-cadherin, N-cadherin and fibronectin (FN) were detected by western blotting in the indicated MCF-7 cells (band intensity was quantified using GAPDH as a normalizer). **C**. Transwell assay wase performed to assess the migration and invasion abilities of the indicated engineered MCF-7 cells. **D**. Relative lnc-408 levels in different BC cell lines were determined by qRT-PCR. (**P*<0.05, ***P*<0.01, ****P*<0.001.).

**Supplemental Figure 2. Knocking out Lnc-408 suppress the invasion of primary BC cells**

**A**. The truncated PCR product of Lnc-408 genomic DNA from a representative single clone with KO Lnc-408 of PL-BC-05. **B**. Relative lnc-408 RNA levels in PL-BC-05 cells with sh Lnc-408 or KO Lnc-408 by qRT-PCR. **C**. Transwell assay was performed to assess the migration and invasion abilities of the indicated engineered PL-BC-05 cells (Scale bars, 100 μm). (**P*<0.05, ***P*<0.01, ****P*<0.001).

**Supplemental Figure 3. Supplemental data for relative miRNAs**

**A**. The ratio of lncRNA-408 in the nucleus and cytoplasm was determined by qRT-PCR. **B**. Survival analysis of patients with BC in the hsa-miR-654-5p high and low expression groups (data source: kmplot breast cancer METABRIC database, n=1262). **C**. Correlation between lnc-408 and hsa-miR-654-5p levels in 60 clinical BC tissues (Pearson correlation test). **D**. Relative lnc-408 and LIMK1 RNA expression levels and LIMK1 protein levels in PL-BC-05 cells with sh Lnc-408, KO Lnc-408 and mimics miR-654-5p. **E**. Correlation between LIMK1 RNA and hsa-miR-654-5p levels in 60 clinical BC tissues (Pearson correlation test). (**P*<0.05, ***P*<0.01, ****P*<0.001).

**Supplemental Figure 4. LIMK1 maintains actin cytoskeletal organization via the p-cofilin/F-actin axis**

**A.** The protein levels of LIMK1, p-cofilin and cofilin were determined by western blotting in engineered Hs578T cells (Hs578T/sh LIMK1 and Hs578T/sh NC), PL-BC-05 cells (PL-BC-05/sh LIMK1 and PL-BC-05/sh NC) and MCF-7 cells (MCF-7/LIMK1 and MCF-7/vec) (band intensity was quantified using GAPDH as a normalizer). **B.** Transwell assay to assess the migration and invasion abilities of the indicated engineered PL-BC-05 cells (Scale bars, 100 μm). **C.** The indicated engineered Hs578T, PL-BC-05 and MCF-7 cells were stained with TRITC phalloidin and DAPI. F-actin is shown under a fluorescence microscope (F-actin was stained red, and nuclei were stained blue; scale bars, 50 μm). (**P*<0.05, ***P*<0.01, ****P*<0.001.).

**Supplemental Figure 5. Correlation between the expression of LIMK1 and MMP2, ITGB1 or COL1A1 in patients with BC**

**A**. Correlation between LIMK1 and MMP2 mRNA levels in BC tissues (Pearson correlation test, an overview of TCGA BRCA data (N=1085) provided by GEPIA). **B**. Correlation between LIMK1 and MMP2 mRNA levels in 60 clinical BC tissues (Pearson correlation test). **C.** Correlation between lnc-408 RNA and MMP2 mRNA levels in 60 clinical BC tissues (Pearson correlation test). **D**. Correlation between LIMK1 and MMP2 IHC scores in 60 clinical BC tissues (Pearson correlation test). **E**. Representative images of LIMK1, MMP2, ITGB1 and COL1A1 expression in two BC tissue samples checked by IHC (Scale bars, 50 μm). **F**. Western blotting to evaluate the protein expression of LIMK1, p-CREB, CREB and MMP2 in engineered BC cells (band intensity was quantified using GAPDH as a normalizer). **G**. IF staining of p-CREB in the engineered PL-BC-05 cells described above, captured using fluorescence microscopy (p-CREB was stained red, and nuclei were stained blue with DAPI (Scale bars, 50 μm). **H**. Western blotting to evaluate the protein expression of LIMK1, p-CREB, CREB, MMP2, ITGB1 and COL1A1 in the designated PL-BC-05 cells (band intensity was quantified using GAPDH as a normalizer). (**P*<0.05, ***P*<0.01, ****P*<0.001.).

**Supplemental Figure 6. Correlation between the expression of LIMK1 and ITGB1, COL1A1 in patients with BC**

**A-I**, **B-I**. Correlation between LIMK1 and ITGB1 or COL1A1 mRNA levels in BC tissues (Pearson correlation test, an overview of TCGA BRCA data (N=1085) provided by GEPIA). **A-II**, **B-II**. Correlation between LIMK1 and ITGB1or COL1A1 mRNA levels in 60 clinical BC tissues (Pearson correlation test). **A-III, B-III.** Correlation between lnc-408 RNA levels and ITGB1 mRNA or COL1A1 mRNA levels in 60 clinical BC tissues (Pearson correlation test). **A-IV**, **B-IV**. Correlation between LIMK1 and ITGB1or COL1A1 IHC scores in 60 clinical BC tissues (Pearson correlation test).

**Supplemental Figure 7. Lnc-408 promotes BC cell invasion via LIMK1**

**A**. qRT-PCR was used to detect Lnc-408 or LIMK1 expression in engineered PL-BC-05 cells (PL-BC-05/sh Lnc-408, PL-BC-05/sh Lnc-408/LIMK1 and PL-BC-05/control; PL-BC-05/KO Lnc-408, PL-BC-05/KO Lnc-408/LIMK1 and PL-BC-05/control). **B**. IF staining of p-CREB in the engineered PL-BC-05 cells described above, captured using fluorescence microscopy (p-CREB was stained red, and nuclei were stained blue with DAPI (Scale bars, 50 μm). **C**. The protein expression levels of LIMK1, p-CREB, CREB, MMP2, ITGB1 and COL1A1 in the indicated PL-BC-05 cells were evaluated by western blotting (band intensity was quantified using GAPDH as a normalizer). **D.** The protein levels of LIMK1, p-cofilin and cofilin were determined by western blotting in engineered Hs578T cells (Hs578T/sh lnc-408, Hs578T/sh lnc-408/LIMK1, and Hs578T/control), engineered PL-BC-05 cells described above and MCF-7 cells (MCF-7/lnc-408, MCF-7/lnc-408/sh LIMK1, and MCF-7/control) (band intensity was quantified using GAPDH as a normalizer). (**P*<0.05, ***P*<0.01, ****P*<0.001.).

**Supplemental Figure 8. Lnc-408 promotes BC cell invasion via LIMK1**

**A.** The indicated engineered Hs578T, PL-BC-05 and MCF-7 cells were stained with TRITC phalloidin and DAPI. F-actin is shown under a fluorescence microscope (F-actin was stained red, and nuclei were stained blue; scale bars, 50 μm). **B**. Transwell assays were performed to assess the migration and invasion abilities of the indicated engineered PL-BC-05 cells (Scale bars, 100 μm). (**P*<0.05, ***P*<0.01, ****P*<0.001.).
