# Supplementary material for: Long non-coding RNA Lnc-408 promotes invasion and metastasis of breast cancer cell by regulating LIMK1
Source: Oncogene. 2021 Jun 2;40(24):4198–213. doi: 10.1038/s41388-021-01845-y (PMC8211561; doi:10.1038/s41388-021-01845-y)
Supplement: Supplementary file 8 — Supplementary Methods and Materials [file 41388_2021_1845_MOESM8_ESM.docx]

**Supplementary Materials and methods**

**RNA microarray analysis**

LncRNA and mRNA microarray analysis was performed as described previously ^[16]^. Total RNA was extracted from secondary generation mammospheres derived from MCF-7/Vector and MCF-7/Twist cells and BC tissue from patients with or without lymphatic metastasis. More than 20,000 lncRNAs and 40,000 cDNA were analyzed using Agilent lncRNA and mRNA arrays (4×180K). Dysregulated lncRNAs or mRNAs were identified as those with a fold change of greater than 2.0.

**RNA extraction and qRT-PCR**

Total RNA was extracted with TRIzol reagent (TAKARA, Japan) and reverse transcribed into cDNA using the PrimeScript RT Reagent Kit (TAKARA, Japan) according to the manufacturer’s instructions. Nuclear and cytoplasmic RNA was extracted using the Thermo PARISTM Kit (Thermo Fisher Scientific, USA) according to the manufacturer’s instructions. Quantitative PCR was performed using SYBR Premix Ex Taq II (TAKARA, Japan) for triplicate cDNA samples, and the average threshold cycle (Ct) values were calculated. The relative expression of target RNA was normalized to β-actin as 2^Ct(β-actin) - Ct(cDNA)^. All primers used in this study were synthesized by Sangon (Shanghai, China), and the sequences are listed in Supplementary Table 1 (for testing lncRNA and mRNA) and Supplementary Table 4 (for testing miRNA).

**Plasmids, mimics and inhibitors**

Synthetic short hairpin RNA (shRNA) oligonucleotides were inserted into the pGLVH1/GFP+Puro vector. The shRNA and negative control (NC) shRNA sequences are listed in Supplementary Table 3. The lnc-408 and LIMK1 sequences were queried from Ensembl (asia.ensembl.org) and inserted into the pGLVCMV/Neo vector, and corresponding infective lentiviruses were constructed and acquired from GenePharma (Shanghai, China). All the mimics and inhibitors used in this research were purchased from GenePharma (Shanghai, China). The procedures for cell transfection with lentivirus, mimics and inhibitors were performed according to the instructions provided by GenePharma.

**TRITC phalloidin staining**

The working solution of TRITC phalloidin (Solarbio, Beijing, China) was prepared in PBS (pH=7.4) at a final concentration of 120 nM. Cells were fixed with 4% paraformaldehyde solution for 10 min. The cells were permeabilized with 0.5% Triton X-100 (Sigma-Aldrich, USA) for 5 min and blocked with 1% BSA in PBS for 40 min at 37 °C. After staining with TRITC phalloidin for 30 min in the dark, cells were stained with 100 nM DAPI (Solarbio, Beijing, China) for 30 s and mounted for examination under fluorescence microscopy (Olympus, Tokyo, Japan).

**Chromatin immunoprecipitation assay**

The chromatin immunoprecipitation (ChIP) assay was performed following the instructions of the SimpleChIP® kit (CST, MA, USA). In brief, formaldehyde was used to crosslink proteins to DNA and stopped using glycine. Cells were collected by centrifugation, resuspended in ChIP Sonication Cell Lysis Buffer + PIC, and then sonicated with proper pulses to fragment chromatin. Ten microlitres of diluted chromatin was removed into a new microfuge tube for use as the input sample. Lysates from separate samples were incubated with immunoprecipitating antibody overnight at 4 °C with rotation, and then protein G magnetic beads were added to each IP reaction and incubated for 2 h at 4 °C with rotation. After washing the magnetic beads, the DNA-protein complex was eluted, and the cross-links were reversed. The purified DNA was resuspended in TE buffer for qRT-PCR. The primers for the indicated promoters are listed in Supplementary Table 5.

**Immunohistochemistry (IHC) and scoring**

Immunohistochemistry was conducted as previously described ^[45]^. Briefly, after dewaxing, dehydration and blocking endogenous peroxidase and nonspecific binding sites, the tissue sections were incubated with rabbit antibodies against LIMK1 (1:200, Abcam), MMP2 (1:200, Abcam, UK), ITGB1 (1:300, Abcam, UK) or COL1A1 (1:200, CST, USA). The images were taken using a Nikon Eclipse 80i microscope (Eclipse 80i, Tokyo, Japan), and the IHC score was assigned based on the proportion score and intensity of the staining, as described previously ^[46]^.

**Primary Breast Cancer Cell Cultural**

Primary breast cancer cell lines were obtained from the tumor tissues of breast cancer patient with surgery in the First Affiliated Hospital of Chongqing Medical University. The tumor tissue was isolated from vascular and fibroadipose tissues, and was cut into small pieces which were resuspended in RPMI 1640 medium with 20% heat-inactivated fetal bovine serum (Gibco) and maintained in a humidified incubator with 37 °C, 5% CO_2_. Primary cells were observed daily and fibroblast cells were removed under sterile condition. After 10 times of passage the primary cells grew well and fast. qRT-PCR was performed to check the relative expression level of Lnc-408. PL-BC-05 showed the highest level of Lnc-408 compared to other cell lines, and was chosen for the further experiments.

**CRISPR/Cas9 Knockout System**

CRISPR/Cas9 knockout system was used to delete Lnc-408. sgRNAs were designed by online tool <http://crispr.dfci.harvard.edu/SSC/> and sequences are listed in Supplementary Table 3. Each end of the Lnc-408 sequence was targeted by two sgRNAs. The sgRNAs were inserted into the px333 vector and corresponding infective lentiviruses were constructed and acquired from GenePharma (Shanghai, China). The PL-BC-05 cells were infected by lentiviruses and selected by G418 for two weeks. Cells survived were diluted into 96-well plates for single cell cloning. The genomic DNA was isolated and the knockout of Lnc-408 was confirmed by PCR (primers are listed in Supplementary Table 1). qRT-PCR was performed to make sure no expression of Lnc-408 in the KO cells.
