# Supplementary material for: Long non-coding RNA Lnc-408 promotes invasion and metastasis of breast cancer cell by regulating LIMK1
Source: Oncogene. 2021 Jun 2;40(24):4198–213. doi: 10.1038/s41388-021-01845-y (PMC8211561; doi:10.1038/s41388-021-01845-y)
Supplement: Supplementary file 9 — ethic permission [file 41388_2021_1845_MOESM9_ESM.pdf]

The Ethics Committee of

ChongQing Medical University

Approval Notice of research paper

Principal Investigators: Yixuan Hou

Title of Projects: Long non-coding RNA Lnc-408 promotes invasion and metastasis of breast cancer cell by regulating LIMK1

Date Submitted: May 1, 2018

Date Reviewed: May 8, 2018

Date Approved: May 15, 2018

The Ethics Committee of Chongqing Medical University has reviewed the proposed use of human subjects in the above-mentioned projects. It is recognized that the rights and the welfare of the subjects are adequately protected; the potential risks are outweighed by potential benefits. The species, strains, grade, specification and number of the animals to be used are justified. Appropriate animal care throughout the experiment, including anesthetics, sedatives should be used. Disposition of animals at end of study euthanasia criteria and method is accordance with the code of practice for the care and use of animals for scientific purposes. We approve papers resulting from the project.

The Ethics Committee of Chongqing Medical University
